# TEPAPA: a novel in silico feature learning pipeline for mining prognostic and associative factors from text-based electronic medical records

**DOI:** 10.1038/s41598-017-07111-0

**Published:** 2017-07-31

**Authors:** Frank Po-Yen Lin, Adrian Pokorny, Christina Teng, Richard J. Epstein

**Affiliations:** 1 Department of Oncology, St Vincent’s Hospital & The Kinghorn Cancer Centre, Darlinghurst, NSW Australia; 2 Garvan Institute of Medical Research, Darlinghurst, NSW Australia; 3 Department of Medical Oncology, Liverpool Hospital, Liverpool, Sydney, NSW Australia

## Abstract

Vast amounts of clinically relevant text-based variables lie undiscovered and unexploited in electronic medical records (EMR). To exploit this untapped resource, and thus facilitate the discovery of informative covariates from unstructured clinical narratives, we have built a novel computational pipeline termed *T*ext-based *E*xploratory *P*attern *A*nalyser for *P*rognosticator and *A*ssociator discovery (TEPAPA). This pipeline combines semantic-free natural language processing (NLP), regular expression induction, and statistical association testing to identify conserved text patterns associated with outcome variables of clinical interest. When we applied TEPAPA to a cohort of head and neck squamous cell carcinoma patients, plausible concepts known to be correlated with human papilloma virus (HPV) status were identified from the EMR text, including site of primary disease, tumour stage, pathologic characteristics, and treatment modalities. Similarly, correlates of other variables (including gender, nodal status, recurrent disease, smoking and alcohol status) were also reliably recovered. Using highly-associated patterns as covariates, a patient’s HPV status was classifiable using a bootstrap analysis with a mean area under the ROC curve of 0.861, suggesting its predictive utility in supporting EMR-based phenotyping tasks. These data support using this integrative approach to efficiently identify disease-associated factors from unstructured EMR narratives, and thus to efficiently generate testable hypotheses.

## Introduction

The widespread digitisation of clinical data through the adoption of electronic medical records (EMR) have speculated many secondary uses across clinical and research applications^1–4^. In particular, as data sharing frameworks have been developed, healthcare data analytics has emerged as a new field of translational science^3^. As an illustrative example in oncology, the CancerLinQ framework of American Society of Clinical Oncology provides a “rapid learning health system” that connects isolated EMR systems across institutions to expedite collaborative patient management^5, 6^. Developing pragmatic, automated methods to leverage this huge resource would soon impact on translational cancer research^7, 8^. Moreover, from a precision medicine perspective, finding accurate associative and prognostic factors should empower clinicians to tailor effective treatments.

Many EMR-based secondary analyses have correlated outcomes data to structured variables (e.g., laboratory and medication) or administrative coding (e.g., billing) to unearth knowledge that would otherwise remain occult^9–13^. These abridged data, however, represent only a proverbial tip of the clinical iceberg. For example, EMR narratives generate great informatic potency via the rich combination of subjective patient encounters with objective and/or measurable clinical events^14–16^. Methods of simple text search and natural language processing (NLP) have been applied to infer patient characteristics (i.e., EMR-based case detection and phenotyping methods) from clinical narratives to discover new and possibly causal associations^13, 17–22^. However, although these high-throughput analyses may be powerful in quantifying the degree of association, an important limitation is that the covariates yet to be recognised by domain experts cannot be reliably assessed.

Hence, to systematically identify unrecognised covariates at an early phase of discovery, we hypothesise a need to mine EMR matrix features in a “deep-data” manner to complement population-based “big-data” inquiries. To this end we present here an unbiased feature-learning pipeline, *T*ext-based *E*xploratory *P*attern *A*nalyser for *P*rognosticator and *A*ssociator discovery (TEPAPA), which combines semantic-free NLP methods, pattern search, and a “pattern-wide association study” (thereafter PatWAS) to capture conserved patterns of EMR text associated with clinical outcomes of interest. With translational utility in mind, TEPAPA is designed to deliver “white-box” interpretable results to researchers for rapid hypothesis generation, thereby providing an open-source framework that drives integration of external NLP and machine learning methods.

To determine how TEPAPA performs in a real-life discovery task, we conduct here a single-centred validation study to determine whether or not clinicopathologic factors associated with human papilloma virus (HPV)-related head and neck squamous cell carcinoma (HNSCC) can be discovered from routine clinical EMR data. The epidemic increases of HPV-related cases over the last two decades reflect changes in sexual practice among younger adults^23^; since the clinicopathologic characteristics associated with this cancer have been thoroughly studied^24–36^, testing of these data sets for rediscovery evaluations is attractive. Beyond this knowledge discovery task, we also examine whether the highly-correlated text features extracted by TEPAPA can be used to classify a patient’s HPV status in combination with supervised machine learning – and if so, to yield a demonstration of practical utility of this pipeline for supporting EMR-based phenotyping applications.

## Methods

### The in silico discovery pipeline

#### Case identification, EMR retrieval, and data cleaning

**Figure 1:**
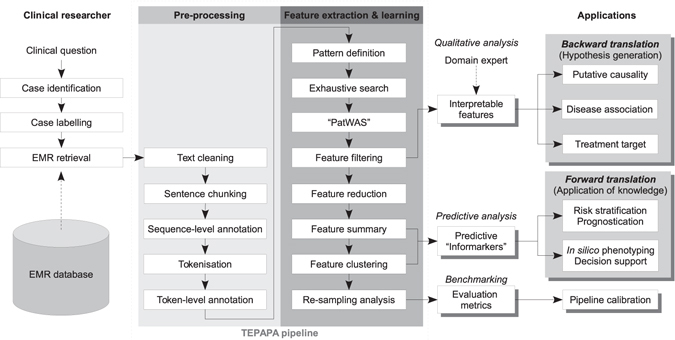
The TEPAPA discovery pipeline. Abbreviations: EMR: electronic medical record.

The discovery process begins with identification of representative cases and controls providing sufficient data quantity and quality to frame a clinical question of interest. Each case is labelled with an outcome variable of interest (either binary or numeric) for correlative analyses. The corresponding EMR text narratives, including clinical correspondence, consultation notes, radiology and pathology reports, are extracted. Sentence chunking is then performed, followed by zero or more annotation methods (see below) prior to transformation into sequences of word-based tokens delimited by white spaces and punctuation marks. The flowchart of analysis is shown in Fig. 1.

#### Text annotation

**Figure 2:**
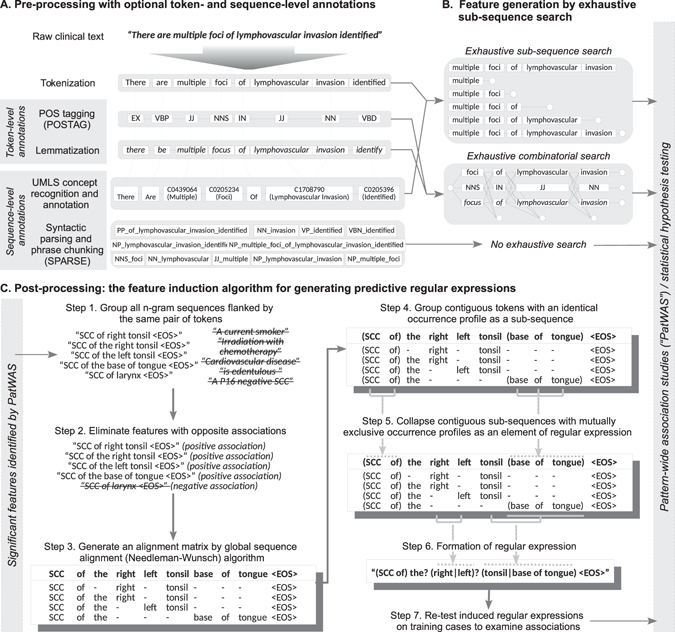
Illustrated methods of annotation, sub-sequence search, and regular expression induction. EMR narratives are tokenized, annotated, and transformed into text fragments (n-gram) prior to association testing. Syntactically similar n-grams are then (optionally) grouped into regular expressions with the aim to aggregate conceptually similar features improve overall recall.

Two classes of optional pre-processing methods were used to annotate the EMR text (Fig. 2A):

1. A *token-level annotation* method that assigns tags to a token in order to reflect its properties. Annotations of this class include labelling of cardinal numbers, word stemming (STEM)^37^, part-of-speech tagging (POSTAG)^38^ and/or lemmatisation. The overall goal here is to improve sensitivity (i.e., recall) of a pattern.
2. A *sequence-level annotation* method that improves specificity through reduction of spurious discoveries by grouping consecutive token descriptors of a given concept into a new token. For example, “*head of pancreas*” is treated as a unigram instead of separate words “*head*”, “*of*”, and “*pancreas*” - which have different meanings. Two annotation methods of this category were examined:
  - *Syntactic parsing* (SPARSE), which transforms a sentence into the PennTree bank format using the Stanford CoreNLP Parser^39^ and new tokens are generated by traversing through each node of the tree structure;
  - *Vocabulary-based concept recognition* maps recognised text fragments into a new unigram based on United Medical Language System (UMLS) vocabulary (Metathesaurus, version 2016AA) using longest-string matching^40, 41^.

#### Feature generation through exhaustive sequence search

The most basic feature for discovery is defined as a string of word-based tokens (*n*-gram). Unique *n*-grams are identified through a corpus-wide exhaustive search (Fig. 2B) and all *n*-grams are used as *binary features* (i.e. either present or absent in a case) in the subsequent association analysis. The extent of search is delimited by sentence and document boundaries. If a token-based annotation method is used, a combinatorial search method is applied to generate all possible sub-sequences using all tokens and tags (Fig. 2B); these patterns are then used in the subsequent association analysis.

The *numeric features*, which take form of “*A* 〈*NUMBER*〉 *B*” (e.g. “*contains* 〈*NUMBER*〉 *metastatic nodes*”, are first identified by extracting all cardinal numbers from the text, followed by identification of a pair of flanking *n*-grams (*A* and *B*) using the same exhaustive search methods above. If a flanking pair occurs more than once in a case, the pattern is discarded to avoid ambiguity. The numeric value is then extracted for association analysis.

#### Statistical association analysis (“PatWAS”)

Non-parametric univariate methods are applied to assess the statistical independence between a feature and the outcome variable of interest. For a binary feature, we first determined a vector to indicate its occurrences across all case (i.e. occurrence profile), followed by calculation of the odds ratio (OR) and Fisher’s exact test for binary outcome variables, and the area under the receiver operating characteristic curve (AUC) for numeric variables (Mann-Whitley-Wilcoxon test). For a numeric feature, the degree of association is determined by AUC (binary outcomes) and Spearman’s ρ (for numeric outcomes).

#### Feature filtering and reduction

Features are filtered by an *ad hoc* significance threshold assigned by the investigator, considering the data characteristics and multiple hypothesis testing. Highly-correlated patterns that do not improve interpretability of results are removed: a feature is removed if there exists a longer sequence sharing the same occurrence profile (e.g., “*extensive liver metastases*” has more explanatory power than “*liver metastases*” and “*metastases*”, if all three *n*-grams appear in the same occurrence profile).

#### Post-processing of binary features by predictive regular expression induction

Syntactically-similar but weakly predictive text fragments may be grouped together to form a stronger “meta-feature” to improve recall. As an example, “*extensive bone metastasis*” and “*extensive liver metastasis*” may be combined to form a regular expression “*extensive* (*bone|liver*) *metastasis*” to indicate a new composite concept. To generate such regular expressions, we first identify all *n*-grams sharing the same starting and ending tokens. Needleman-Wunsch algorithm is then applied to perform global sequence alignment, followed by a consolidation algorithm to group sequences into a linear, non-recursive expression as depicted in Fig. 2C. Previously, regular expressions have been shown to improve precision in information extraction from clinical text^42^. In contrast to the local alignment approach^42^, we used global alignment because a wildcard at the either end of a regular expression would result in non-discriminant matching of token and the consequent loss of specificity. The degree of association of induced regular expression is then reassessed by the PatWAS step above.

#### Performance considerations

Heuristics are applied to reduce the hypothesis space as “curse of dimensionality” is unavoidable in any high-dimensional analyses. Techniques used to improve the pipeline efficiency include aggressive result caching, token indexing, and search termination if an elongating pattern occurs only once in the corpus. In particular, exhaustive traversal through all annotated subclasses (e.g. part of speech and concept hierarchy) would incur a theoretical time complexity of O(c^N^) (c > 1, i.e. exponential time), thus needing aggressive feature reduction: when a token-based annotation method is used, we first remove annotations that are uniquely associated with a token without an occurrence elsewhere in the corpus; up to 90% of annotations may be removed by this approach.

### The HNSCC validation cohort

#### Study population

Consecutive patients presented to a tertiary referral hospital over a twelve-month period (February 2015–February 2016) were screened for inclusion. The cases were dichotomised into HPV-related and -unrelated groups by documented *in situ* hybridisation (ISH) results (either mentioned in correspondence or pathology report) or P16 (cyclin-dependent kinase inhibitor 2A protein, encoded by *CDKN2A* gene) immunohistochemistry (at least 2+), which was used as a surrogate marker if an ISH assay was not performed.

#### Data extraction

The free-text component of clinical documents associated with each case, including multidisciplinary team (MDT) meeting reports, clinic letters, radiology and pathology reports, were extracted from EMR to form the corpus. The patient identifiers, name and role of clinicians, and practice addresses were removed using string matching, followed by a manual verification by the lead investigator. Three investigators independently reviewed the HPV status of all cases (FL, AP, and CT). Blood-based assays were not included in this analysis.

#### Statistical and exploratory analyses

Clinicopathologic variables were analysed by descriptive statistics using R statistical environment version 3.3. Qualitative analyses of pattern discovered by TEPAPA was reviewed by the authors and also compared with published literature.

#### Predictive analysis

We further examined whether the highly-associated text patterns can be used in conjunction with supervised learning to predict case labels. To assess how pipeline variations may affect the accuracy of prediction and computational time, we used a factorial design to vary methods of annotation (part-of-speech tagging, syntactic parsing, word stemming, UMLS-based token aggregation), post-processing (with or without regular expression induction), threshold selection (log_10_ deviation from best threshold), in conjunction with different machine learning algorithms.

Each pipeline was applied to identify text features associated with the HPV status. To avoid selecting highly co-linear features, we applied hierarchical clustering with Unweighted Paired Groups Mean Average (UPGMA) algorithm and Euclidean distance to cluster the features into one-tenth of sample size (i.e. N/10) groups. The features with the smallest p-value from each group were used for classification. Waikato Environment for Knowledge Analysis (WEKA) 3.6.6 was used for classifier training and evaluation^43^. Both generative (logistic regression, LR) and two discriminative classifiers Naive Bayes (NB) and alternating decision tree model (ADTree)^44^ with ten boosting iterations were examined. The predictive accuracy was assessed by AUC averaging over 25 bootstrap runs. The relative computational time was also analysed. Multiple linear regressions were used for the statistical analysis.

#### Ethics approval and informed consent

This study was approved by St. Vincent’s Hospital Human Research Ethics Committee (HREC), Sydney, Australia. Data collection and analysis were conducted in accordance to the HREC regulations and the National Statement on Ethical Conduct in Human Research (2007), published by the Australian National Health and Medical Research Council (NHMRC). The need for informed consent was waived by the HREC for this retrospective study.

### Ethics

This study was approved by the Human Research Ethics Committee (HREC) of St. Vincent’s Hospital, Sydney, Australia (Reference number: LNR/15/SVH/458).

## Results

### Characteristics of the study cohort and EMR corpus

**Figure 3:**
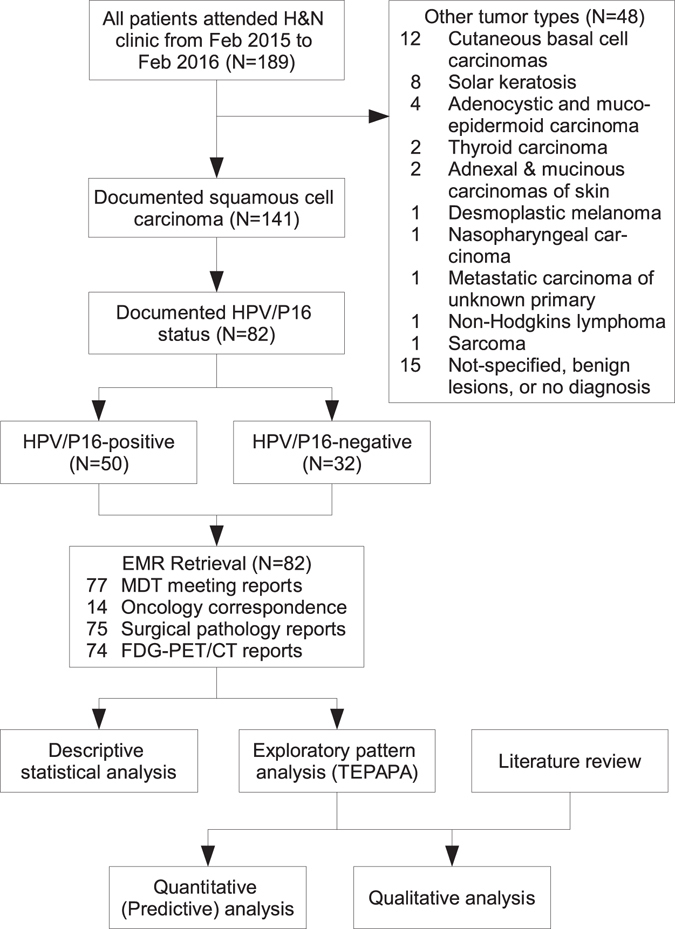
Flowchart of data analysis of the validation dataset.

One-hundred-and-eighty-nine consecutive patients attended the head and neck multidisciplinary team (MDT) cancer clinic at the study site from February 2015 to February 2016 were screened (Table 1). A total of 141 patients with documented squamous cell carcinoma were further inspected (Fig. 3). Approximately two thirds (N = 50) of 82 patients had documented HPV/P16 positive diseases (i.e. HPV-related) either in the pathology report or in other clinical correspondence (e.g., performed by external pathology services). Three cases were subsequently found to contain no tumour in subsequent surgical or repeated biopsy specimens.

**Table 1:** The characteristics of HNSCC cohort by HPV/P16 status.

Characteristic	Value	HPV/P16 status				P^*a*^
		Positive (n = 50)		Negative (n = 32)		
		N	(%)	N	(%)	
*Demographics*						
Age at diagnosis	Mean (years)	61.5	(95%CI: 58.9–64.2)	65.5	(95%CI: 60.9–70)	0.14
Gender	Male	44	(88)	25	(78)	0.38
	Female	6	(12)	7	(22)	
*Tumour characteristics*						
Diagnosis	Squamous cell carcinoma	49	(98)	30	(94)	0.28
	Other tumour types	1	(2)	2	(6)	
Laterality	Right	20	(61)	4	(40)	0.37
	Left	12	(36)	6	(60)	
	*Not specified* ^*b*^	17		22		
Site of origin	Oropharynx	42	(89)	14	(48)	<0.01^*c*^
	Skin	2	(4)	3	(10)	
	Larynx	0	(0)	9	(31)	
	Lip	1	(2)	2	(7)	
	Nasal cavity	1	(2)	0	(0)	
	Nasopharynx	1	(2)	0	(0)	
	Salivary gland	0	(0)	1	(3)	
	*Not specified*	3		3		
Recurrent disease	Yes	20	(43)	14	(45)	1
	No	26	(57)	17	(55)	
	*Not specified*	4		1		
*Anatomical stage*						
T category	T1	11	(23)	7	(23)	0.52
	T2	14	(29)	5	(16)	
	T3	14	(29)	10	(32)	
	T4	3	(6)	5	(16)	
	Tx	6	(12)	4	(13)	
	*Not specified*	2		1		
N category	N0	10	(21)	11	(35)	0.35
	N1	9	(19)	6	(19)	
	N2, nos	3	(6)	1	(3)	
	N2a	7	(15)	1	(3)	
	N2b	11	(23)	7	(23)	
	N2c	7	(15)	2	(6)	
	N3	0	(0)	1	(3)	
	Nx	1	(2)	2	(6)	
	*Not specified*	2		1		
M category	M0	43	(90)	28	(90)	0.39
	M1	0	(0)	1	(3)	
	Mx	5	(10)	2	(6)	
	*Not specified*	2		1		
TNM Stage (7th edition)	I	2	(4)	5	(17)	0.17
	II	2	(4)	2	(7)	
	III	13	(27)	7	(23)	
	IV	31	(65)	16	(53)	
	*Not specified*	2		2		
*Smoking status*						
Ever smoked	Yes	22	(56)	20	(74)	0.23
	No	17	(44)	7	(26)	
	*Not specified*	11		5		
Smoking history	Median (pack-years)	0	(IQR: 0–27.5)	25	(IQR: 0–50)	0.02
	*Not specified*	19		8		
Current smoker	Yes	11	(28)	10	(37)	0.625
	No	28	(72)	17	(63)	
	*Not specified*	11		5		
Current amount	Median	0	(IQR: 0–0)	10	(IQR: 0–22.5)	0.17
	*Not specified*	28		17		
Last smoked	Median (years ago)	1.12	(IQR: 0.812–3.19)	21	(IQR: 18.5–24)	0.02
	*Not specified*	50		26		
*Alcohol use*						
Ever consumed	Yes	27	(82)	21	(84)	1
	No	6	(18)	4	(16)	
	*Not specified*	17		7		
Current drinker	Yes	23	(70)	18	(72)	1
	No	10	(30)	7	(28)	
	*Not specified*	17		7		
Current amount	Median (grams/day)	60	(IQR: 20–80)	40	(IQR: 20–80)	0.70
	*Not specified*	23		11		

NB: IQR: Inter-quartile range; (a) Fisher’s exact test was used for hypothesis testing on categorical and binary data. Shapiro-Wilk test was used to determine the normality for numeric data. One-way Analysis of Variance (ANOVA) and Kruskal-Wallis tests were used to determine the difference between means (normally-distributed) and median (non-normally distributed) data respectively. (b) Significant between-group difference (p < 0.05) on the number of missing values (c) Statistically significant at α = 0.01.

The discovery corpus consisted of five types of clinical text: (1) MDT meeting reports (N = 77), (2) correspondence from medical oncology clinic (N = 14), (3) anatomical pathology reports (N = 75), and (4) radiology reports of 18F-fluorodeoxyglucose Positron Emission Tomography/Computed Tomography (FDG-PET/CT, N = 74), (5) All of the above clinical text (N = 82) including other non-cancer-specific EMR, including correspondence from other specialties, non-oncology radiology reports, administrative records).

### Qualitative analyses of text features associated with HPV/P16 status in HNSCC patients

#### Exploratory analysis of MDT meeting reports

The top binary feature (text fragment) associated with HPV-related HNSCC was “*base of*” (OR: 10.5, p = 4.1 × 10^−5^, pattern S2a.1) which was part of the phrase “*base of tongue*”. This was followed by “*the right tonsil*” (OR: 22.9, p = 0.0012, S2a.2), “*M0*,” (OR: 20.5, p = 0.0023, S2a.3), and “*positive*” (OR: 5.7, p = 0.0029, S2a.4), which were indicative of disease site, stage, and part of phrase “*HPV/P16 positive*” respectively. The full list of patterns is described in Table S2a.

After application of regular expression induction algorithm, the list became more informative. Regular expressions describing the site of disease (e.g. “*the* (*right|left*)*? base of tongue*”, S2c.2 and “*SCC of the right* (*tonsil|base of tongue|glossotonsillar sulcus*) *-*”, S2c.9), treatment modality (S2c.7), and HPV/P16 status (S2c.17) were discovered. A phrase describing the most likely disease stage in HPV-related cases (“(*T3 N2c|T1 N2b|cT1 N2a*) *M0*”, i.e. non-metastatic disease with low T- but high N-stage) was identified at a more liberal filtering threshold (OR: 11.9, p = 0.038).

Text features associated with HPV-unrelated disease were also extractable from the MDT meeting reports (Table S2e–h). At a first glance, the majority of unigrams was not seemingly interpretable. However, a close examination of the corpus text showed that these words were either part of a conserved expression or words embedded within a group of concepts. For instance, the word “*management*” (S2e.1) referred to a number of phrases describing upfront surgery (e.g. “*Initial management will require*… *dissection*”, “*Initial management* … *surgical*”, 4 of 7 cases). The word “*than*” was associated with concept of ever-consumed alcohol (part of “*consumed less/more than x gram of alcohol*”, S2e.2). The fragment “*disease with*” (S2e.6) was part of phrases “*ischaemic heart disease with*…” (N = 4) and “*peripheral vascular disease with*…” (N = 2), indicating a composite concept of advanced atherosclerotic disease. Again, the induction of regular expression produced more interpretable concepts than simple *n*-gram fragments (Table S2f and g).

**Figure 4:**
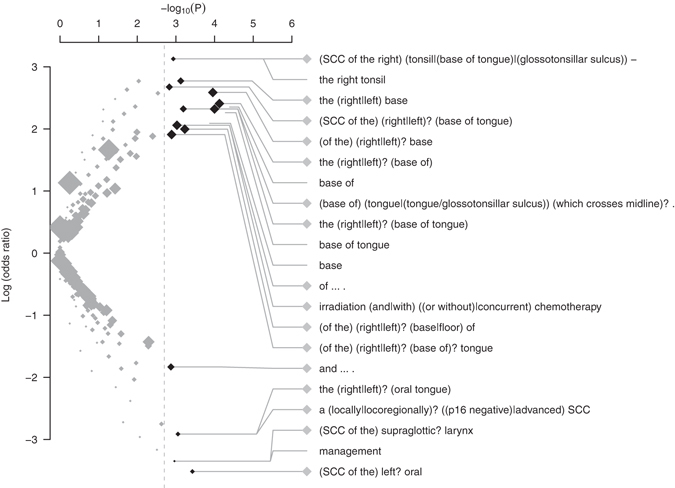
Volcano plot showing the ranking text features associated with HPV status discovered from the HNSCC MDT reports. Note: Labels of patterns with p < 0.002 are shown in this plot. Legend: ◆: regular expression. ∙: n-gram text fragments. The pattern of regular expression “(*A|B*)” indicates either *A* or *B* would match the string, and “?” indicates an optional element. The size of diamond or circle is proportional to total number of cases mentioning the text patterns in the EMR.

The volcano plot is shown in Fig. 4, and a list of informative patterns is summarised in Table 2.

**Table 2:** Informative features associated with HNSCC by HPV status as discovered by TEPAPA.

Log (OR)	P	N	Text feature	Type	EMR Source	Interpretation	Crossref.
**Informative features associated with HPV-related HNSCC**							
3.50	3.0 × 10^−6^	25	“HPV (studies|genotypes|status):? P16 immunohistochemistry:? Positive”	R	Pathology	HPV status (Self-referent)	(S3c.1)
3.89	6.2 × 10^−6^	20	“HPV (positive|genotypes: Positive|associated squamous cell carcinoma|related).”	R	Pathology	HPV status (Self-referent)	(S3c.2)
3.29	2.0 × 10^−5^	23	“No FDG avid? pulmonary (nodules|nodule) or pleural”	R	PET	(Lack of) metastasis to the lung	(S4c.1)
3.14	5.6 × 10^−5^	21	“HPV related”	N	Pathology	HPV status (Self-referent)	(S3b.6)
2.06	0.00094	24	“irradiation (and|with) (or without|concurrent) chemotherapy”	R	MDT	Management	(S2c.7)
2.76	0.0093	9	“oropharyngectomy:”	N	Pathology	Management, site of primary tumor	(S3a.22)
3.23	0.0011	13	“SCC of the right (tonsil|base of tongue|glossotonsillar sulcus) -”	R	MDT	Site of primary tumor	(S2d.4)
2.68	0.0015	16	“SCC of the (right|left)? base of tongue”	R	MDT	Site of primary tumor	(S2d.5)
3.02	0.0023	11	“M0”	N	MDT	Stage	(S2a.3)
2.89	0.0047	10	“non-keratinising”	N	Pathology	Pathology feature	(S3a.16)
2.77	0.0092	9	“p16? positive,? HPV? positive”	R	MDT	HPV status (Self-referent)	(S2c.17)
**Informative features associated with HPV-unrelated HNSCC**							
−3.54	0.00035	8	“for decalcification”	N	Pathology	Pathology feature	(S3e.2)
−2.91	0.00089	10	“a (locally|locoregionally)? (p16 negative|advanced) SCC”	R	MDT	HPV status and pathology feature	(S2h.3)
−3.17	0.0031	6	“SCC of the supraglottic? (lower lip|larynx).”	R	MDT	Site of primary tumor	(S2g.7)
−2.96	0.0086	5	“likely to? require adjuvant radiation therapy”	R	MDT	Management	(S2g.10)
−3.35	0.0011	7	supportive care	N	MDT	Management	(S2f.3)
−2.59	0.0058	8	“differentiated, keratinising squamous cell carcinoma”	N	Pathology	Pathology feature	(S3e.23)
−2.59	0.0058	8	“well differentiated”	N	Pathology	Pathology feature	(S3e.26)

Note: The type field indicates the type of text features (N: n-gram fragments or R: regular expression). N indicates number of documents containing the text features. Abbreviations: Log (OR): Log odds ratio. MDT: Multidisciplinary team meeting.

#### Exploratory analysis on other sub-corpora

The analysis of pathology reports identified text fragments describing the results of HPV/P16 assay as the ranking feature (“: *Positive*” and “*: Negative*”, S3a.1 and S3e.1), among other relevant factors (Tables 2 and S3). Likewise, the sites of primary tumour (S3a.2–4) and the associated concepts (e.g. “*for decalcification*”, S3e.2, indicating the need to process bony surgical specimen for microscopic examination, thus less likely to be at an oropharyngeal site) were also identified. In the FDG-PET/CT reports, we found conflicting results describing abnormal pulmonary nodules where two phrases describing both the presence and absence of associations with lung metastasis were found (e.g. S4c.1 and S4c.7). Further examinations of the EMR text showed that the negation qualifiers were not captured due to lexical variations (e.g. “not” or “no evidence of”), and the negative concepts appeared to be more conserved in its expression. An analysis of oncology correspondences did not yield statistically significant entries at α = 0.025.

### Qualitative comparison of discovered concepts with epidemiological literature

A practical measure of quality of discovery is to compare the algorithmically discovered concepts against published literature (Table 3). In this analysis, our pipeline consistently recovered concepts associated with primary tumour site, the commonest anatomical staging at presentation, and the primary treatment modality in association with a patient’s HPV status. Indirect associations of cigarette and alcohol exposure, cardiovascular comorbidities were also described. From the pathology reports, TEPAPA identified histological grade, non-keratinising epithelium, morphology, and lack of epithelial dysplasia as features correlated to HPV-related disease. While patients with HPV-related disease are known to have a more favourable prognosis^36^, survival data was not available for examination. Sexual and marijuana history were not recorded in the EMR, and comorbidities were also inconsistently documented.

**Table 3:** Literature-based comparison of features associated with HNSCC by HPV status.

Variables	HPV status		Examples of highly-ranked, informative features	Reference
	HPV-related	HPV-unrelated	Log(OR), P-value (Crossref.)	
**Demographics**				
Age	Younger	Older	(*Not identified*)	24
Married	Associated	NS	(*Not consistently documented in EMR*)	25
**Social History**				
Cigarette and alcohol exposure	Associated	Strongly associated	“**than**” *Log(OR) = −2.75, P = 0.0024 (S2e.2)	24–26
Marijuana use	Associated	Associated	(*Not documented in EMR*)	25
Poor oral hygiene (incl. tooth loss)	Not associated	Associated	“**is (…|a restored dentition|…|edentulous|…)**.” ^†^ Log(OR) = −1.43, P = 0.0051 (S2h.7)	25, 26
**Sexual history**				
Oral sex partners	Associated	NS	(*Not documented in EMR*)	24–27, 29
Number of lifetime sexual partners	Associated	NS	(*Not documented in EMR*)	25, 27, 29
**Comorbidities**				
Cardiovascular	Risk factors (e.g. Hypertension)	Macrovascular arthrosclerotic disease	“**disease with**” Log(OR) = −3.2, P = 0.0031 (S2e.6)*	25
Primary tumor site	Oropharynx	Non-oropharynx	“**SCC of the right (tonsil|base of tongue|glossotonsillar sulcus)**” - Log(OR) = 3.23, P = 0.0011(S2d.4) “**SCC of the (right|left)? base of tongue**” Log(OR) = 2.68, P = 0.0015 (S2d.5)	24, 26, 28, 30–32
**Anatomical stage**				
T stage	Early T-stage		“**M0**,” Log(OR) = 3.02 p = 0.002 (S2a.3) “**((T3 N2c)|(T1 N2b)|(cT1 N2a)) M0**” Log(OR) = 2.48, P = 0.038 “**a large single lymph node exhibiting metastatic cystic? moderately differentiated non-keratinising? squamous cell carcinoma**”. Log(OR) = 2.89, P = 0.0047(S3d.53)	33
Nodal status	Multilevel, “High N-stage” Cystic nodes			24, 30, 33, 34
**Pathology features**				
Grade	Moderately to poorly differentiated	Moderately differentiated	“**non-keratinising**” Log(OR) = 2.9, P = 0.0047 (S3a.16), “(**poorly differentiated|non-keratinizing|non-keratinising|focally keratinizing)? squamous cell carcinoma**” Log(OR) = 2.67 P = 0.0015 (S3c.35) **of (…|basaloid type/Non-keratinizing|…)**. ^†^Log(OR) = 2.87, P = 0.00062 (S3c.19) “**with (high|low)? (grade|mild) dysplasia**” Log(OR) = −3.37, P = 0.001(S3g.11)	26, 30
Keratinisation	Absent	Present		26
Other features	Basaloid morphology	Epithelial dysplasia		26, 28, 32
**Management**				
Locally advanced disease (T3/4 or N2/3)	Surgery + adjuvant radiotherapy +/− concurrent chemotherapy		“**irradiation (and|with) (or without|concurrent) chemotherapy**” Log(OR) = 2.06, P = 0.00094 (S2c.7)	35
**Treatment outcome**				
Overall survival	Better prognosis	Poorer prognosis	(*Not assessable by this dataset*)	36

Abbreviations: NS: Not significant. Log(OR): Log odds ratio; Note: *Refers to part of “*consumed* (*greater|less*) *than*”, which was a phrase used to describe “ever-consumption of alcohol”. ^†^The index concept was revealed only through “overfitting” the concept to a regular expression pattern flanked by two tokens. See main text for detailed discussions.

We have found that the regular expression induction algorithm can meaningfully group closely related concepts together if they are flanked by a pair of highly specific phrases (e.g. “*SCC of* … *base of tongue*”, S2d.5), but less so if the flanking texts are made up of common words. For instance, the concepts related to poor oral hygiene (“*restored dentition*” and “*edentulous*”) were admixed with other unrelated concepts (S2h.7) as a result of overfitting the training data to non-specific text pattern “*of* …. ”.

### Binary and numeric features associated with other clinical variables

Exploratory analyses of other clinicopathologic variables were performed to demonstrate the generalisability of method (Table S5). The pipeline found the phrases “*He*” (p = 1.7 × 10^−13^, S5.1) and “*She is*” (p = 1.6 × 10^−15^, S5.3) being associated with patient’s gender. The age of patient was associated with mentions of “*chronic*” (AUC: 0.75, p = 0.00011, S5.7) and “*retired*” (AUC: 0.76, p = 0.00025, S5.8). Elderly patients were more likely to have a chest X-ray performed with an anterior-posterior projection (AUC: 0.85, p = 7.6 × 10^−6^, S5.6), suggesting a more complicated post-operative course in this population. Descriptors of recurrent cases were recovered (S5.18–21). Regular expressions describing nodal status, which were explainable by phrases summarizing the extra-nodal spread (S5.13 and S5.15), nodal stage (S5.14), and the phrase “*there is no lymphadenopathy*” (S5.17) were identified. A conserved regular expression associated with smoking status was found (e.g., “*a cigarette/heavy/current smoker*”, S5.29). Ever-smokers were characterised by the regular expression “*a* (*reformed*)*? cigarette/heavy/current smoker*” (the question mark denotes an optional word, S5.36). Current users of alcohol were associated with the use of a quantification phrase “*g of alcohol daily*” (S5.38). Phrases associated with patients who have never consumed alcohol have also been identified (S5.39).

The age at diagnosis was perfectly correlated to a structured numeric field in the MDT report recording the patient’s age (p = 1.4 × 10^−37^, S6.1). The maximum Standardised Uptake Value (SUVMax) of a lesion on FDG-PET/CT was negatively associated with advanced age (ρ = −0.69, p = 0.00087, S6.3). The amount of alcohol consumed by the patient was also extractable (S6.4). The HPV-related cases were more likely to have higher localised SUVMax values (S6.8). Smoking cessation was associated with the phrase “〈*number*〉 *pack*” (p = 3.3 × 10^−5^, S6.6).

### Phenotyping of HPV/P16 status using features learned from EMR text

With all sub-corpora included, the HPV/P16 status could be classified with an overall AUC of 0.861 using EMR narratives alone. While a relationship between the parameters and accuracy was not distinct, the type of text and filtering threshold appeared to be important (Table 4 and Figure S8). As expected, pathology reports, the most likely sub-corpus containing HPV/P16 status, topped among the four sub-corpora. Multiple regression analysis suggested that sequence-level annotation, stemming, and UMLS annotation were more likely to yield an improved performance (except for FDG-PET/CT reports). For predictions based on pathology reports, Naive Bayes was numerically superior to ADTree, although in general the performance was comparable across classifiers. Regular expression induction did not improve accuracy in more specialised sub-corpora. The combinatorial search methods (POSTAG and SPARSE) were unable to complete at the predefined resource limit for bootstrapping analysis when the entire corpus was used for discovery.

**Table 4:** Predictive performance by varying methods annotation type, threshold selection, and machine learning methods.

Pipeline variations	Corpus type									
	MDT meeting reports (N = 77)		Oncology letters (N = 14)		Pathology reports (N = 75)		FDG-PET/CT reports (N = 74)		All inclusive (N = 82)	
	Est.	P	Est.	P	Est.	P	Est.	P	Est.	P
Mean (Intercept)	0.634		0.559		0.835		0.759		0.861	
Annotation method										
None	(Ref.)									
POSTAG	0.006	0.13	0.031	<0.001	−0.043	<0.001	−0.062	<0.001	NA	
STEM	0.010	0.009	0.011	0.05	0.005	0.08	0.017	<0.001	0.013	0.059
SPARSE	−0.017	<0.001	0.056	<0.001	0.004	0.17	−0.005	0.32	NA	
UMLS	0.013	<0.001	0.030	<0.001	0.004	0.17	−0.190	<0.001	0.014	<0.001
Post-processing										
None	(Ref.)									
REGEXI	−0.003	0.17	0.003	0.44	−0.003	0.09	−0.002	0.50	0.007	0.018
Machine learning algorithm										
ADTree	(Ref.)									
Logistic regression	−0.0002	0.94	0.015	<0.001	−0.007	0.006	−0.003	0.38	−0.017	<0.001
Naive Bayes	0.005	0.10	0.018	<0.001	0.018	<0.001	0.003	0.38	0.006	0.126
Threshold selection										
Optimal threshold	(Ref.)									
−*log*_10_ deviation from the optimal threshold	−0.022	<0.001	−0.040	<0.001	−0.013	<0.001	−0.011	<0.001	0.003	0.15
*Adjusted R* ^*2*^	0.40		0.65		0.66		0.85		0.72	

NB: Abbreviations: ADTree: Alternating decision tree (10-boosting iterations); FDG-PET/CT:18F-fluorodeoxyglucose Positron Emission Tomography/Computed Tomography; MDT: multidisciplinary team; POSTAG: Part-of-speech tagging with word lemmatization; REGEXI: regular expression induction algorithm; SPARSE: syntactic parsing; STEM: token-level annotation by word stemming using Snowball algorithm; UMLS: sequence-level annotation using Meta-thesaurus from the United Medical Language System (UMLS) version 2016 AA.

An empirical observation was made such that the computational time was linearly correlated to the corpus size (in characters, r^2^ = 0.994, p = 0.0002), conforming to linear time complexity [O(N)]. Annotation with word stemming, part-of-speech tagging, and syntactic parsing generally increased training time, whereas UMLS-based token aggregation generally reduced the computational time (Table S7). Variations in the filtering threshold and regular expression induction both produced comparable time usage across different text types.

## Discussion

The central finding of this research is that clinically relevant associative knowledge is discoverable from EMR text by combining semantic-free NLP methods with association analysis. Our method sensitively identifies key clinicopathologic factors that differentiate subgroups of HNSCC patients by HPV status. Hence, we expect our approach to find useful signals associated with clinical outcomes in other domain. This tool provides an adjunct for efficiently generating new hypotheses guiding downstream investigations for as-yet-unsolved biomedical problem scenarios.

This work also highlights the possibility of finding plausible associations using only a relatively small cohort of routinely-collected EMR patient data. Most factors associated with virally-implicated HNSCC have been found through EMR retrieved from a single site. However, the selection of relevant corpus appeared to be important; for example, we found no significant association factors from oncologic correspondence. The lack of association was not unexpected because of the small corpus size, as well as the fact that chemotherapy is only a subsidiary modality for managing non-metastatic HNSCC^35^. Current guidelines also do not yet recommend a different treatment regimen for HPV-related disease, despite speculations for de-intensification in this population^35, 36^.

Several strengths of our feature generation and ranking approach suggest useful applications. First, TEPAPA extracts knowledge in the form of clear text and its derivatives, which allows direct transformation of these patterns into searchable formats. The “white-box” approach is advantageous because it allows domain experts to rapidly generate hypotheses and to re-identify contextual information about a case when discrepancies arise, as shown in our analysis. Second, the PatWAS method addresses the “cognitive gaps” which occur at the time of designing an observational study. The unbiased method avoids the problem where a researcher focuses only on a set of familiar variables for testing in an *ad hoc* manner, thereby permitting discovery of novel associations. This approach is attractive because most EMR data contain unstructured narratives, and the key concepts may only be described by using non-standardised lexicons. Third, the backbone of our method assumes no underlying knowledge, and thus is expected to work on other biomedical texts, whether formal (e.g. MEDLINE abstracts) or informal (e.g., social network data), to support discovery in distinct settings. Fourth, TEPAPA can find predictive, text-based “informarkers” to allow risk stratification, support *in silico* phenotyping tasks, and extract information from EMR. The feasibility of this integrative approach is supported by our predictive analysis.

One capability of TEPAPA is to aggregate syntactically similar text fragments into regular expressions to aid data interpretability. In our classification task, however, inclusion of regular expressions did not consistently improve accuracy over that obtained using “bag of token” features alone. Consistent with previous studies, regular expressions provided only a small performance benefit over use of simple word vectors in classification tasks, bearing a weak but correlative trend to the training sample size^42, 45^. Accordingly, methods that aggregate text fragments (as in induction of regular expressions) – although generating features with better sensitivity (recall) - provide little overall additional information when used in conjunction with a multivariate learner for classification and prediction.

**Figure 5:**
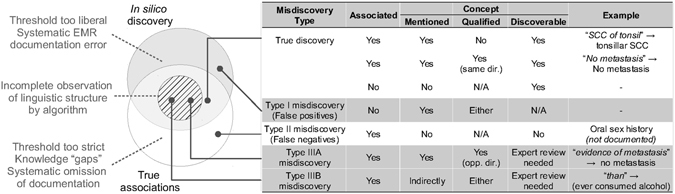
Scenarios, examples, and potential sources of misdiscovery.

Although our method appears to provide useful insights into EMR data, the results still need to be scrutinised by domain experts referring back to the original text. To better understand this limitation, we categorised three scenarios of misdiscovery, each of which has a unique characteristic with potential solutions (Fig. 5). Both types I (false positives) and II misdiscoveries (false negatives) can be affected by inappropriate threshold assignment during the feature filtering step. Moreover, type II misdiscovery can result from insufficient information in the corpus. For instance, sexual history was not recorded in our dataset, and was therefore unable to be discovered computationally. Systematic omissions such as this represent an absolute limitation for all types of EMR-based discovery. Type III misdiscovery (wrongly positive) described two related subgroups (incorrect qualifier assignment, IIIA and partial correlated patterns, IIIB). Both problems arise from the algorithm failing to fully examine the underlying semantic structure, resulting in only partial observations. Such misdiscovery represents the ceiling of capability for semantic-free NLP methods, but could be amendable to a richer knowledge representation by incorporating a comprehensive semantic analysis on platforms such as MedLEE^45^ and cTAKES^46^ during the pre-processing step. A trend was evident from our analysis which suggested that a more sophisticated representation (e.g., regular expression) confers better descriptive power (e.g., versus *n*-grams). Incorporating contextual knowledge is thus expected to improve the quality of machine-generated features by considering the linguistic structure more fully.

Several challenges for future research are clear. First, the optimal method for selecting an objective filtering threshold remains unsolved, as the exhaustive search algorithms guarantee the generation of patterns that are not identically and independently distributed. As such, the conventional methods for adjusting for multiple hypothesis testing, such as Bonferroni^47^ and Benjamini-Hochberg corrections^48^, would be unable to identify a suitable cut-off. Second, as in all high-dimensional analysis, overfitting may occur if a pattern is over-calibrated to fit the training data. Incorporating ensemble selection with early-stopping may avoid building an overly-complex model^49^. Third, the caveats of epidemiological research (e.g. biases and confounders) still apply, and asking a relevant clinical question remains paramount. Fourth, downstream of plausible text pattern identification, rigorous confirmatory studies remain necessary before drawing a definitive clinical conclusion; EMR-based analyses inherently suffer from bias, noise, missing data, and inconsistency^50–53^. Fifth, features extracted by TEPAPA are presented in conventional statistical quantities that are widely accepted by the clinical community (e.g., odds ratio, AUC, and p-value). While this application-oriented approach may help to generate new hypotheses for clinical research, alternative feature selection algorithms and regularised variable regression methods (e.g., elastic net)^54^ may be better suited to select patterns for building multivariate models for classification. More research is thus needed to identify how to best combine feature generation and selection methods in the context of clinical text classification. Last but not least, meticulous removal of patient identifiers is required to avoid inadvertent breaches of patient privacy, particularly in a data-sharing environment.

In conclusion, we have developed a novel computational pipeline for systematically identifying hitherto-unrecognised covariates from EMR narratives through associative text-mining analyses. Our results support the clinical and translational research use of TEPAPA and its future derivatives in efficiently extracting *de novo* knowledge and hypotheses from EMR in the background.

### Data Availability

The source code of TEPAPA can be obtained from http://tepapadiscoverer.org/.

## Electronic supplementary material

Supplementary Information
