# Supplementary material for: TEPAPA: a novel in silico feature learning pipeline for mining prognostic and associative factors from text-based electronic medical records
Source: Sci Rep. 2017 Jul 31;7:6918. doi: 10.1038/s41598-017-07111-0 (PMC5537364; doi:10.1038/s41598-017-07111-0)

Supplementary Material

TEPAPA: a novel *in silico* feature learning pipeline for mining prognostic and associative factors from text-based electronic medical records

Frank Lin [1,2*], Adrian Pokorny [3], Christina Teng [4], Richard J Epstein [1,2]

[1] Department of Oncology, St Vincent's Hospital & The Kinghorn Cancer Centre, NSW, Australia
[2] Garvan Institute of Medical Research, Darlinghurst NSW Australia
[3] The Chris O'Brien Lifehouse, Sydney, Camperdown, NSW Australia
[4] Department of Medical Oncology, Liverpool Hospital, NSW Australia

* Corresponding author

List of contents

Table S1. Characteristics of the corpus used in main study

Table S2. Full list of features listed mined by TEPAPA (MDT reports)

Table S3. Full list of features listed mined by TEPAPA (Anatomy pathology reports)

Table S4. Full list of features listed mined by TEPAPA (FDG PET/CT reports)

Table S5. Full list of features mined by TEPAPA – other variables

Table S6. Numeric features mined by TEPAPA

Table S7. Relative computational time by type of features included

Figure S8. Average predictive performance by classifier and feature filtering threshold

Table S1. Characteristics of the corpus used in the main study

Variable	Metric	Corpus type				
		**MDT Report** (N=77)	**Medical oncology clinic Letters** (N=14)	**Anatomical pathology reports** (N=75)	**FDG-PET & Radiology reports** (N=74)	**All inclusive** (N=82)
HPV/P16 status	Positive	46 (60%)	6 (43%)	45 (60%)	45 (61%)	50 (61%)
File size (lines)	corpus	5,106	1,220	22,312	10,588	77,278
	Per case	66	87	298	143	942
File size (bytes)	corpus	146,112	47,795	870,635	399,482	2,727,529
	Per case	1,898	3,414	11,609	5,398	33,263
Words	corpus	21,879	6,850	125,352	57,390	373,539
	Per case	284	489	1,671	776	4,555

Note: MDT: Multidisciplinary meeting.

Table S2. Full list of features listed mined by TEPAPA (MDT reports)

Table S2(a): Positive binary features (n-gram) without sequence-level annotation

Log (OR)	P	N	Pattern	Comments	Crossref.
2.35	4.1×10^{-5}	32	**base of**	SITE (Type IIIB MD)	(S2a.1)
3.13	0.0012	12	**the right tonsil**	SITE	(S2a.2)
3.02	0.0023	11	**M0 ,**	STAGE	(S2a.3)
1.74	0.0029	25	**positive**	HPV STATUS (Type IIIB MD)	(S2a.4)
2.90	0.0046	10	**glossotonsillar sulcus**	SITE	(S2a.5)

Table S2(b): Positive binary features (n-gram) with sequence-level annotation using UMLS vocabulary

Log (OR)	P	N	Pattern	Comments	Crossref.
2.35	4.1×10^{-5}	32	**base of tongue**	SITE	(S2b.1)
2.08	0.00027	29	**positive**	HPV STATUS (Type IIIB MD)	(S2b.2)
3.13	0.0012	12	**the right tonsil**	SITE	(S2b.3)
3.13	0.0012	12	**sulcus**	SITE (Type IIIB MD)	(S2b.4)

Table S2(c): Positive binary features (regular expressions) without sequence-level annotation

<table>
<tr><th>Log (OR)</th><th>P</th><th>N</th><th>Pattern</th><th>Comments</th><th>Crossref.</th></tr>
<tr><td>2.41</td><td>7.5×10^{-5}</td><td>28</td><td>the (right|left)? base of</td><td>SITE (Type IIIB MD)</td><td>(S2c.1)</td></tr>
<tr><td>2.32</td><td>0.0001</td><td>27</td><td>the (right|left)? base of tongue</td><td>SITE</td><td>(S2c.2)</td></tr>
<tr><td>2.59</td><td>0.00011</td><td>24</td><td>of the (right|left)? base</td><td>SITE (Type IIIB MD)</td><td>(S2c.3)</td></tr>
<tr><td>2.00</td><td>0.00059</td><td>28</td><td>of the (right|left)? (base|floor) of</td><td>SITE (Type IIIB MD)</td><td>(S2c.4)</td></tr>
<tr><td>2.32</td><td>0.00064</td><td>21</td><td>base of (tongue|tongue.glossotonsillar sulcus) which crosses midline? .</td><td>SITE</td><td>(S2c.5)</td></tr>
<tr><td>2.77</td><td>0.00075</td><td>17</td><td>the (right|left) base</td><td>SITE (Type IIIB MD)</td><td>(S2c.6)</td></tr>
<tr><td>2.06</td><td>0.00094</td><td>24</td><td>irradiation (and|with) (or without|concurrent) chemotherapy</td><td>MANAGEMENT</td><td>(S2c.7)</td></tr>
<tr><td>2.06</td><td>0.00094</td><td>24</td><td>of (tongue|subclavicular disease)? (which crosses midline|tongue.glossotonsillar sulcus)? .</td><td>SITE</td><td>(S2c.8)</td></tr>
<tr><td>3.13</td><td>0.0012</td><td>12</td><td>SCC of the right (tonsil|base of tongue| glossotonsillar sulcus) -</td><td>SITE</td><td>(S2c.9)</td></tr>
<tr><td>1.91</td><td>0.0013</td><td>27</td><td>of the (right|left)? base of? tongue</td><td>SITE</td><td>(S2c.10)</td></tr>
<tr><td>2.68</td><td>0.0015</td><td>16</td><td>SCC of the (right|left)? base of tongue</td><td>SITE</td><td>(S2c.11)</td></tr>
<tr><td>2.57</td><td>0.0029</td><td>15</td><td>SCC of the (right|left) tonsil</td><td>SITE</td><td>(S2c.12)</td></tr>
<tr><td>2.57</td><td>0.0029</td><td>15</td><td>the left? base of tongue .</td><td>SITE</td><td>(S2c.13)</td></tr>
<tr><td>1.88</td><td>0.004</td><td>22</td><td>irradiation (and|with) concurrent</td><td>MANAGEMENT</td><td>(S2c.14)</td></tr>
<tr><td>2.77</td><td>0.0092</td><td>9</td><td>the (right|left)? glossotonsillar sulcus</td><td>SITE</td><td>(S2c.15)</td></tr>
<tr><td>2.77</td><td>0.0092</td><td>9</td><td>right (tonsil|IIA|base of tongue) ,</td><td>SITE</td><td>(S2c.16)</td></tr>
<tr><td>2.77</td><td>0.0092</td><td>9</td><td>, p16? positive ,? HPV? positive</td><td>HPV STATUS</td><td>(S2c.17)</td></tr>
<tr><td>2.77</td><td>0.0092</td><td>9</td><td>involving the (right|left)? base</td><td>SITE (Type IIIB MD)</td><td>(S2c.18)</td></tr>
</table>

Table S2. Full list of features listed mined by TEPAPA (MDT reports) (Cont'd)

Table S2(d): Positive binary features (regular expressions) with sequence-level annotation using UMLS vocabulary

<table>
<tr><th>Log (OR)</th><th>P</th><th>N</th><th>Pattern</th><th>Comments</th><th>Crossref.</th></tr>
<tr><td>2.59</td><td>0.00011</td><td>24</td><td>of the (right|left)? base of tongue</td><td>SITE</td><td>(S2d.1)</td></tr>
<tr><td>2.77</td><td>0.00075</td><td>17</td><td>the (right|left) base of tongue</td><td>SITE</td><td>(S2d.2)</td></tr>
<tr><td>2.06</td><td>0.00094</td><td>24</td><td>irradiation (and|with) (or without|concurrent) chemotherapy</td><td>MANAGEMENT</td><td>(S2d.3)</td></tr>
<tr><td>3.23</td><td>0.0011</td><td>13</td><td>SCC of the right (tonsil|base of tongue|glossotonsillar sulcus) -</td><td>SITE</td><td>(S2d.4)</td></tr>
<tr><td>2.68</td><td>0.0015</td><td>16</td><td>SCC of the (right|left)? base of tongue</td><td>SITE</td><td>(S2d.5)</td></tr>
<tr><td>2.57</td><td>0.0029</td><td>15</td><td>the (level II|left glossotonsillar sulcus|base of tongue|left tonsil) -</td><td>NODAL, SITE</td><td>(S2d.6)</td></tr>
<tr><td>1.88</td><td>0.004</td><td>22</td><td>irradiation (and|with) concurrent</td><td>MANAGEMENT</td><td>(S2d.7)</td></tr>
<tr><td>2.77</td><td>0.0092</td><td>9</td><td>the (right|left)? glossotonsillar sulcus</td><td>SITE</td><td>(S2d.8)</td></tr>
<tr><td>2.77</td><td>0.0092</td><td>9</td><td>right (tonsil|IIA|base of tongue) ,</td><td>SITE</td><td>(S2d.9)</td></tr>
<tr><td>2.77</td><td>0.0092</td><td>9</td><td>, p16? positive ,? HPV? positive</td><td>HPV STATUS</td><td>(S2d.10)</td></tr>
<tr><td>2.77</td><td>0.0092</td><td>9</td><td>involving the (right|left)? base of tongue</td><td>SITE</td><td>(S2d.11)</td></tr>
</table>

Table S2(e): Negative binary features (n-gram) without sequence-level annotation

Log (OR)	P	N	Pattern	Comments	Crossref.
-3.35	0.0011	7	**management**		(S2e.1)
-2.75	0.0024	9	**than**		(S2e.2)
-2.75	0.0024	9	**radiation therapy .**	MANAGEMENT	(S2e.3)
-2.75	0.0024	9	**larynx**	SITE	(S2e.4)
-3.17	0.0031	6	**SCC of the oral tongue**	SITE	(S2e.5)
-3.17	0.0031	6	**disease with**		(S2e.6)
-3.17	0.0031	6	**supportive care**	MANAGEMENT	(S2e.7)
-2.57	0.0061	8	**less**		(S2e.8)
-2.57	0.0061	8	**tissues**		(S2e.9)
-2.57	0.0061	8	**posterior**	TUMOUR LOCATION	(S2e.10)
-2.57	0.0061	8	**of his**		(S2e.11)
-1.75	0.0068	15	**anterior**	TUMOUR LOCATION	(S2e.12)
-2.96	0.0086	5	**MRI scan shows**		(S2e.13)
-2.96	0.0086	5	**care .**		(S2e.14)
-2.96	0.0086	5	**is currently**		(S2e.15)
-2.96	0.0086	5	**laryngeal**		(S2e.16)
-2.96	0.0086	5	**partial**		(S2e.17)
-2.96	0.0086	5	**progressive**		(S2e.18)

Table S2. Full list of features listed mined by TEPAPA (MDT reports) (Cont'd)

Table S2(f): Negative binary features (n-gram) with sequence-level annotation using UMLS vocabulary

Log (OR)	P	N	Pattern	Comments	Crossref.
-2.91	0.00089	10	**oral tongue**	SITE	(S2f.1)
-3.35	0.0011	7	**management**		(S2f.2)
-3.35	0.0011	7	**supportive care**	MANAGEMENT	(S2f.3)
-2.75	0.0024	9	**larynx**	SITE	(S2f.4)
-2.57	0.0061	8	**Anterior**	TUMOUR LOCATION	(S2f.5)
-2.57	0.0061	8	**posterior**	TUMOUR LOCATION	(S2f.6)
-2.96	0.0086	5	**MRI scan shows**		(S2f.7)
-2.96	0.0086	5	**grade**		(S2f.8)
-2.96	0.0086	5	**is currently**		(S2f.9)

Table S2(g): Negative binary features (regular expressions) without sequence-level annotation

Log (OR)	P	N	Pattern	Comments	Crossref.
-3.52	0.00037	8	**SCC of the left? oral**	SITE (Type IIIB MD)	(S2g.1)
-2.91	0.00089	10	**the (right|left)? oral tongue**	SITE	(S2g.2)
-2.91	0.00089	10	**a (locally|locoregionally)? (p16 negative| advanced) SCC**	HPV STATUS	(S2g.3)
-3.35	0.0011	7	**SCC of the supraglottic? larynx**	SITE	(S2g.4)
-1.83	0.0013	21	**and (in excellent general health|neck dissection|nondrinker|well|review|adjuvant radiation therapy|base of tongue| dyslipidaemia|dysphagia for solids|gastro-oesophageal reflux disease|retromolar trigone|IV|ramipril) .**	Mixed concept: COMORBIDITY, MANAGEMENT, PERFORMANCE STATUS, SITE, SOCIAL HISTORY, SYMPTOM	(S2g.5)
-2.75	0.0024	9	**SCC of the left? oral? tongue**	SITE	(S2g.6)
-3.17	0.0031	6	**SCC of the supraglottic? (lower lip|larynx) .**	SITE	(S2g.7)
-1.43	0.0051	39	**is (a restored dentition|with palliative intent| SCC on biopsy|mobile|normal|poor|100 g daily|edentulous|clinically clear|known to the unit|required|intact) .**	Mixed concept: MANAGEMENT, ORAL HYGIENE, SOCIAL HISTORY	(S2g.8)
-1.49	0.0052	23	**He has? (will require|been|works as)? a**		(S2g.9)
-2.96	0.0086	5	**likely to? require adjuvant radiation therapy**	MANAGEMENT	(S2g.10)
-2.96	0.0086	5	**the supraglottic? larynx .**	SITE	(S2g.11)

Abbreviations: MD: Misdiscovery

Table S2. Full list of features listed mined by TEPAPA (MDT reports) (Cont'd)

Table S2(h): Negative binary features (regular expressions) with sequence-level annotation using UMLS vocabulary

Log (OR)	P	N	Pattern	Comments	Crossref.
-3.52	0.00037	8	**SCC of the left? oral tongue**	SITE	(S2h.1)
-3.52	0.00037	8	**SCC of the supraglottic? (oral tongue|larynx|lower lip) .**	SITE	(S2h.2)
-2.91	0.00089	10	**a (locally|locoregionally)? (p16 negative|advanced) SCC**	HPV STATUS	(S2h.3)
-1.91	0.001	19	**and (in excellent general health|neck dissection|nondrinker|review|adjuvant radiation therapy|base of tongue|dyslipidaemia|dysphagia for solids|gastro - oesophageal reflux disease|retromolar trigone|IV|ramipril) .**	COMORBIDITY, MANAGEMENT, PERFORMANCE STATUS, SITE, SOCIAL HISTORY, SYMPTOM	(S2h.4)
-3.35	0.0011	7	**SCC of the supraglottic? larynx**	SITE	(S2h.5)
-2.75	0.0024	9	**left (neck|level II|otalgia|level 1|cheek) .**	NODAL	(S2h.6)
-1.42	0.0048	34	**is (a restored dentition|with palliative intent|SCC on biopsy|mobile|generally fit and well|poor|100 g daily|edentulous|clinically clear|known to the unit|required) .**	MANAGEMENT, ORAL HYGIENE, PERFORMANCE STATUS, SOCIAL HISTORY	(S2h.7)
-2.57	0.0061	8	**the left (neck|level II|level 1|cheek) .**	NODAL	(S2h.8)
-2.96	0.0086	5	**likely to? require adjuvant radiation therapy**	MANAGEMENT	(S2h.9)
-2.96	0.0086	5	**the supraglottic? larynx .**	SITE	(S2h.10)

Note: In Tables S2(c), (d), (g), and (h), regular expressions that group only cardinal numbers as options [e.g. *“size (15|20|30|...) mm”*] are not shown for brevity.

Table S3: Full list of features listed mined by TEPAPA (Pathology reports)

Table S3(a): Positive binary features (n-gram) without sequence-level annotation

Log (OR)	P	N	Pattern	Comments	Crossref.
3.68	4.5×10^{-7}	27	**: Positive**	HPV STATUS (Type IIIB MD)	(S3a.1)
3.14	7.6×10^{-7}	30	**tonsillar**	SITE	(S3a.2)
3.04	2.1×10^{-6}	29	**tongue base**	SITE	(S3a.3)
3.41	7.7×10^{-6}	24	**tonsil**	SITE	(S3a.4)
3.62	4.3×10^{-5}	17	**positive .**		(S3a.5)
3.14	5.6×10^{-5}	21	**HPV related**	HPV STATUS	(S3a.6)
3.53	9.3×10^{-5}	16	**indicate**		(S3a.7)
3.53	9.3×10^{-5}	16	**Positive .**		(S3a.8)
2.87	0.00062	18	**Specimen 9 : Labelled " left**		(S3a.9)
2.06	0.00091	24	**: HPV**	HPV STATUS (Type IIIB MD)	(S3a.10)
2.23	0.0013	20	**lymphoid tissue**		(S3a.11)
1.54	0.0023	39	**carcinoma in**		(S3a.12)
3.01	0.0024	11	**Clinical Information : R**		(S3a.13)
2.89	0.0047	10	**HPV associated**		(S3a.14)
2.89	0.0047	10	**of the largest**		(S3a.15)
2.89	0.0047	10	**, non-keratinising**	PATHOLOGY FEATURE	(S3a.16)
2.89	0.0047	10	**tumour " , the specimen consists of**		(S3a.17)
2.47	0.0057	14	**(0/10**		(S3a.18)
2.47	0.0057	14	**related .**		(S3a.19)
2.76	0.0093	9	**x 30 x 10 mm .**		(S3a.20)
2.76	0.0093	9	**pharyngeal**		(S3a.21)
2.76	0.0093	9	**oropharyngectomy :**		(S3a.22)
2.76	0.0093	9	**associated squamous cell carcinoma**		(S3a.23)
2.76	0.0093	9	**Right oropharyngectomy**		(S3a.24)
1.33	0.0099	34	**dimension**		(S3a.25)

Table S3: Full list of features listed mined by TEPAPA (Pathology reports) – (Cont'd)

Table S3(b): Positive binary features (n-gram) with sequence-level annotation using UMLS vocabulary

Log (OR)	P	N	Pattern	Comments	Crossref.
3.68	$4.5{\times}10^{-7}$	27	**: Positive**	HPV STATUS (Type IIIB MD)	(S3b.1)
3.04	$2.1{\times}10^{-6}$	29	**tongue base**	SITE	(S3b.2)
3.32	$2{\times}10^{-5}$	23	**tonsillar**	SITE	(S3b.3)
3.23	$2.5{\times}10^{-5}$	22	**tonsil**	SITE	(S3b.4)
3.62	$4.3{\times}10^{-5}$	17	**positive .**		(S3b.5)
3.14	$5.6{\times}10^{-5}$	21	**HPV related**	HPV STATUS	(S3b.6)
3.53	$9.3{\times}10^{-5}$	16	**Positive .**		(S3b.7)
3.53	$9.3{\times}10^{-5}$	16	**indicate**		(S3b.8)
2.87	0.00062	18	**Specimen 9 : Labelled " left**		(S3b.9)
2.06	0.00091	24	**: HPV**	HPV STATUS (Type IIIB MD)	(S3b.10)
3.01	0.0024	11	**, non**		(S3b.11)
3.01	0.0024	11	**Clinical Information : R**		(S3b.12)
2.89	0.0047	10	**tumour " , the specimen consists of**		(S3b.13)
2.89	0.0047	10	**HPV associated**		(S3b.14)
2.89	0.0047	10	**of the largest**		(S3b.15)
1.65	0.0055	24	**Metastatic**		(S3b.16)
2.47	0.0057	14	**(0/ 10**		(S3b.17)
2.47	0.0057	14	**related .**		(S3b.18)
2.76	0.0093	9	**oropharyngectomy :**		(S3b.19)
2.76	0.0093	9	**x 30 x 10 mm .**		(S3b.20)
2.76	0.0093	9	**Right oropharyngectomy**		(S3b.21)
2.76	0.0093	9	**base of tongue**	SITE	(S3b.22)
2.76	0.0093	9	**tonsillar lymphoid tissue**	SITE	(S3b.23)
1.33	0.0099	34	**dimension**		(S3b.24)

Table S3: Full list of features listed mined by TEPAPA (Pathology reports) – (Cont'd)

Table S3(c): Positive binary features (regular expressions) without sequence-level annotation

Log (OR)	P	N	Pattern	Comments	Crossref.
3.50	$3{\times}10^{-6}$	25	**HPV (studies|genotypes|status) :? P16 immunohistochemistry :? Positive**	HPV STATUS	(S3c.1)
3.89	$6.2{\times}10^{-6}$	20	**HPV (positive|genotypes : Positive|associated squamous cell carcinoma|related) .**	HPV STATUS	(S3c.2)
3.89	$6.2{\times}10^{-6}$	20	**for (high risk|High)? (HPV genotypes|P16) : Positive**	HPV STATUS	(S3c.3)
3.80	$1.6{\times}10^{-5}$	19	**tongue base (tumour|biopsy)? .**	SITE	(S3c.4)
3.80	$1.6{\times}10^{-5}$	19	**tongue base (tumour|biopsy)? .? ,**	SITE	(S3c.5)
3.71	$2{\times}10^{-5}$	18	**base (of tongue|tumour|biopsy)? . ,**	SITE	(S3c.6)
3.23	$2.5{\times}10^{-5}$	22	**P16 immunohistochemistry? : Positive**	HPV STATUS	(S3c.7)
3.62	$4.3{\times}10^{-5}$	17	**risk (. is positive|HPV genotypes : Positive| probe- Negative) .**	HPV STATUS	(S3c.8)
3.14	$5.6{\times}10^{-5}$	21	**Labelled . (right|left)? lateral? tongue base**	SITE, TUMOUR LOCATION	(S3c.9)
3.14	$5.6{\times}10^{-5}$	21	**: (SCC R tonsil|1 and 2|Positive|See recent path|Reactive lymphoid hyperplasia|Not identified) .**	Mixed concept: SITE, HPV STATUS	(S3c.10)
3.53	$9.3{\times}10^{-5}$	16	**tongue base (tumour|biopsy)? (: No evidence|. , the specimen consists) of**	SITE	(S3c.11)
3.53	$9.3{\times}10^{-5}$	16	**tongue base (tumour|.)? .**	SITE	(S3c.12)
3.43	0.00021	15	**high risk (. is positive|HPV genotypes : Positive) .**	HPV STATUS	(S3c.13)
3.43	0.00021	15	**level (1B|3|4|5A neck dissection|IIa) 8**		(S3c.14)
3.43	0.00021	15	**level (3|4|2A|5A|5B neck dissection|Vb) 9**		(S3c.15)
3.34	0.00046	14	**tumour (. , the specimen consists|invades to a depth|deposit but no evidence) of**		(S3c.16)
3.34	0.00046	14	**: P16 immunohistochemistry :? (positive| Positive) In situ**	HPV STATUS	(S3c.17)
2.01	0.00055	28	**to (lateral|deep|inferior|this|follow|1C|2D|3G) .**	TUMOUR LOCATION	(S3c.18)
2.87	0.00062	18	**of (HPV studies|specimen 1|tissue|all margins|this finding is uncertain|bone| keratinization|fat|basaloid type . non-keratinizing .) .**	PATHOLOGY FEATURE	(S3c.19)
1.83	0.00081	30	**squamous cell carcinoma and squamous cell carcinoma? in**		(S3c.20)
2.06	0.00091	24	**and (2|1B|2B|squamous cell carcinoma in situ| both show cystic degeneration|all embedded Block 1A|3B|anterior|focal acute on chronic inflammation identified|bone|contains brown watery fluid|9D .|neutrophils|1H- epiglottis transverse sections) .**	Mixed concept: PATHOLOGY FEATURE, TUMOUR LOCATION	(S3c.21)
3.23	0.001	13	**P16 immunohistochemistry : (positive| Positive) In situ**	HPV STATUS	(S3c.22)
3.13	0.0012	12	**right (2A|tonsil|oropharyngectomy) .**	SITE	(S3c.23)
3.13	0.0012	12	**, (6|9 o.clock is black|poorly differentiated|16| tonsillar lymphoid tissue|bone|non-keratinising|therefore) ,**	PATHOLOGY FEATURE, SITE	(S3c.24)
3.13	0.0012	12	**HPV (studies indicate the|associated)**	HPV STATUS	(S3c.25)

<table>
<tr><th>Log (OR)</th><th>P</th><th>N</th><th>Pattern</th><th>Comments</th><th>Crossref.</th></tr>
<tr><td></td><td></td><td></td><td>squamous cell carcinoma</td><td></td><td></td></tr>
<tr><td>3.13</td><td>0.0012</td><td>12</td><td>Right lateral? tongue base</td><td>SITE</td><td>(S3c.26)</td></tr>
<tr><td>3.13</td><td>0.0012</td><td>12</td><td>The (HPV studies|results)? indicate the? (squamous cell carcinoma|tumour) is positive for? HPV</td><td>HPV STATUS</td><td>(S3c.27)</td></tr>
<tr><td>3.13</td><td>0.0012</td><td>12</td><td>The (HPV studies|results)? (indicate|true margin|roughened surface) is inked blue and? the</td><td></td><td>(S3c.28)</td></tr>
<tr><td>3.13</td><td>0.0012</td><td>12</td><td>carcinoma (,|is) (positive for|non-keratinising Differentiation.highest grade : Grade 3.poorly differentiated)? HPV</td><td>PATHOLOGY FEATURE, HPV STATUS</td><td>(S3c.29)</td></tr>
<tr><td>3.13</td><td>0.0012</td><td>12</td><td>tonsillar type? lymphoid? tissue and skeletal muscle? .</td><td>SITE</td><td>(S3c.30)</td></tr>
<tr><td>3.13</td><td>0.0012</td><td>12</td><td>tumour (.|present) ,</td><td></td><td>(S3c.31)</td></tr>
<tr><td>3.13</td><td>0.0012</td><td>12</td><td>. right (2A|oropharyngectomy|lateral)? tongue base? .</td><td>SITE, MANAGEMENT, TUMOUR LOCATION</td><td>(S3c.32)</td></tr>
<tr><td>3.13</td><td>0.0012</td><td>12</td><td>Clinical Information : SCC? R</td><td></td><td>(S3c.33)</td></tr>
<tr><td>2.77</td><td>0.0014</td><td>17</td><td>. (Right|Left|left)? tongue base</td><td>SITE</td><td>(S3c.34)</td></tr>
<tr><td>2.67</td><td>0.0015</td><td>16</td><td>, (poorly differentiated|non-keratinizing|non-keratinising|focally keratinizing)? squamous cell carcinoma</td><td>PATHOLOGY FEATURE</td><td>(S3c.35)</td></tr>
<tr><td>1.70</td><td>0.0018</td><td>32</td><td>cell carcinoma and squamous cell carcinoma? in</td><td></td><td>(S3c.36)</td></tr>
<tr><td>3.01</td><td>0.0024</td><td>11</td><td>HPV studies :? P16 immunohistochemistry : (positive|Positive) In situ</td><td>HPV STATUS</td><td>(S3c.37)</td></tr>
<tr><td>3.01</td><td>0.0024</td><td>11</td><td>Block 10A- (one|four)? (lymph|two .lymph)? (nodes|node) bisected? ; 10B-</td><td></td><td>(S3c.38)</td></tr>
<tr><td>3.01</td><td>0.0024</td><td>11</td><td>Positive In situ (hybridisation . Ventana INFORM ISH .|hybridization) for</td><td></td><td>(S3c.39)</td></tr>
<tr><td>3.01</td><td>0.0024</td><td>11</td><td>tonsillar type? lymphoid tissue</td><td>SITE</td><td>(S3c.40)</td></tr>
<tr><td>3.01</td><td>0.0024</td><td>11</td><td>the (squamous cell carcinoma|tumour) is HPV related</td><td>HPV STATUS</td><td>(S3c.41)</td></tr>
<tr><td>3.01</td><td>0.0024</td><td>11</td><td>right (2A|tonsil|oropharyngectomy) . ,</td><td>SITE</td><td>(S3c.42)</td></tr>
<tr><td>3.01</td><td>0.0024</td><td>11</td><td>. (right|left) neck? level 5A</td><td>NODAL</td><td>(S3c.43)</td></tr>
<tr><td>3.01</td><td>0.0024</td><td>11</td><td>P16 (is|immunohistochemistry : Positive|are both|+ve) positive? .</td><td>HPV STATUS</td><td>(S3c.44)</td></tr>
<tr><td>3.01</td><td>0.0024</td><td>11</td><td>: The? (HPV studies|results|Positivity for P53 on immunohistochemistry may) indicate</td><td></td><td>(S3c.45)</td></tr>
<tr><td>3.01</td><td>0.0024</td><td>11</td><td>: (Metastatic|The)? HPV (studies indicate the|associated|related) (moderately|moderate to poorly)? differentiated? squamous cell</td><td>HPV STATUS</td><td>(S3c.46)</td></tr>
<tr><td>3.01</td><td>0.0024</td><td>11</td><td>5B- (four|five) possible? lymph? nodes</td><td></td><td>(S3c.47)</td></tr>
<tr><td>3.01</td><td>0.0024</td><td>11</td><td>, (HPV studies|3|two of which show metastatic moderate|invading) to</td><td></td><td>(S3c.48)</td></tr>
<tr><td>3.01</td><td>0.0024</td><td>11</td><td>tongue base .? :</td><td>SITE</td><td>(S3c.49)</td></tr>
<tr><td>3.01</td><td>0.0024</td><td>11</td><td>squamous cell carcinoma (,|is) positive for? HPV</td><td>HPV STATUS</td><td>(S3c.50)</td></tr>
<tr><td>3.01</td><td>0.0024</td><td>11</td><td>. right (2A|oropharyngectomy|lateral)? tongue base? . ,</td><td>SITE, TUMOUR LOCATION</td><td>(S3c.51)</td></tr>
<tr><td>2.14</td><td>0.0026</td><td>19</td><td>2A (neck dissection|.)? : Metastatic</td><td></td><td>(S3c.52)</td></tr>
<tr><td>2.57</td><td>0.0029</td><td>15</td><td>seven lymph? nodes (.|identified are all negative for malignancy)? .</td><td></td><td>(S3c.53)</td></tr>
</table>

Log (OR)	P	N	Pattern	Comments	Crossref.
2.57	0.0029	15	pieces of (fatty|red|tan)? tissue		(S3c.54)
1.65	0.0033	28	of (three|seven|four|five|eight|twenty one|six|thirteen|seventeen)? lymph nodes		(S3c.55)
1.88	0.0038	22	the (posterior half|centre)? (of the|multiple levels examined .|entire) specimen		(S3c.56)
1.50	0.0043	33	the (tumour deposit but no evidence|largest|junction|area|centre|site|presence|floor|absence|remainder|significance) of		(S3c.57)
1.52	0.0044	30	of (three|seven|a|largest|four|involved|five|eight|six|thirteen|seventeen) lymph		(S3c.58)
2.89	0.0047	10	. (right|left) neck? level 5B	NODAL	(S3c.59)
2.89	0.0047	10	10 :? (.|Labelled)? .? (Left|left) neck		(S3c.60)
2.89	0.0047	10	12 : Labelled .? (right|left)? level? (3|2B)? (.|mm) ,		(S3c.61)
2.89	0.0047	10	The (HPV studies|results)? (indicate|roughened surface is inked blue and) the (squamous cell carcinoma|tumour|specimen) is		(S3c.62)
2.89	0.0047	10	ISH .? for high risk HPV genotypes : Positive	HPV STATUS	(S3c.63)
2.89	0.0047	10	indicate the (squamous cell carcinoma|tumour) is HPV related	HPV STATUS	(S3c.64)
2.89	0.0047	10	: (SCC|Left|.SCC|Suspected) right? tongue base	SITE	(S3c.65)
2.89	0.0047	10	2A (neck dissection|.|+ 3) :		(S3c.66)
2.89	0.0047	10	: (Right|Left|Labelled .) biopsy? right? tonsil	SITE	(S3c.67)
2.89	0.0047	10	the (inferior|other)? (deep|half|false) margin? is inked black	PATHOLOGY & SURGICAL CHARACTERISTICS	(S3c.68)
2.89	0.0047	10	, two of which show metastatic moderate to? (poorly|non-keratinising Differentiation. highest grade : Grade 3. poorly) differentiated	PATHOLOGY FEATURE	(S3c.69)
2.89	0.0047	10	squamous cell carcinoma (,|is) HPV? (present|studies to follow|related) .	HPV STATUS	(S3c.70)
2.89	0.0047	10	pieces of (fatty|red|tan)? tissue ,		(S3c.71)
2.89	0.0047	10	COMMENT : The? (HPV studies|results|Positivity for P53 on immunohistochemistry may) indicate		(S3c.72)
2.89	0.0047	10	in the (posterior half|centre)? (of the|multiple levels examined .) specimen		(S3c.73)
2.89	0.0047	10	fatty tissue ,? 35 x? 25		(S3c.74)
2.89	0.0047	10	Sections show (squamous mucosa with reactive lymphoid|fibrofatty|salivary gland|fragments of granulation|fibrotic|tonsilar) tissue	SITE	(S3c.75)
2.89	0.0047	10	P16 immunohistochemistry : (positive|Positive) In situ hybridisation	HPV STATUS	(S3c.76)
2.89	0.0047	10	2 : Right neck dissection? (level 1B|tonsil|oropharyngectomy) .	NODAL, SITE	(S3c.77)
2.89	0.0047	10	3 : Labelled . right level? 2B? neck dissection? level? (2B|5A|III)? .		(S3c.78)
2.89	0.0047	10	30 x 20 x? 10 mm in aggregate? .		(S3c.79)
2.04	0.0051	18	tumour (is present at|. ,|appears to extend to|invades into) the		(S3c.80)

Log (OR)	P	N	Pattern	Comments	Crossref.
2.04	0.0051	18	**the 3 o.clock? deep? (lateral|excision|surgical)? margin (in|is)? inked blue? (and|from)? the**	SURGICAL CHARACTERISTICS	(S3c.81)
2.04	0.0051	18	**the (specimen consists of a piece of firm|margin is inked blue|anterior|jugular vein|first cystic cavity ; 8E-) and**	SURGICAL CHARACTERISTICS	(S3c.82)
2.04	0.0051	18	**level (1B|4|2A +|Ib|IIb) 3**		(S3c.83)
2.04	0.0051	18	**. (tongue base|lateral|inferior|anterior|medial|superior|Negative|Deep) margin**	Mixed concept: SITE, HPV STATUS, SURGICAL CHARACTERISTICS	(S3c.84)
2.47	0.0057	14	**transverse sections , serially? (embedded|through the centre of the specimen)? from**		(S3c.85)
2.47	0.0057	14	**, (poorly differentiated|non-keratinizing|non-keratinising|focally keratinizing) squamous**	PATHOLOGY FEATURE	(S3c.86)
2.47	0.0057	14	**, (3|6 ,|the specimen consists of a piece of firm|deep margin inked black|skeletal muscle|keratinising) and**	PATHOLOGY FEATURE, SURGICAL CHARACTERISTICS	(S3c.87)
2.47	0.0057	14	**: The? (HPV studies|results)? (tumour invades into|indicate|Approximately 50% of) the**		(S3c.88)
2.47	0.0057	14	**and (the area|one|no evidence|fragments|fibrous clefts in keeping with base) of**	SITE	(S3c.89)
2.47	0.0057	14	**level (3|4|2A|IIa|IIb) 5**		(S3c.90)
2.47	0.0057	14	**studies :? P16 immunohistochemistry : Positive**	HPV STATUS	(S3c.91)
1.79	0.0077	21	**1B (3|6|8|.) .**		(S3c.92)
1.79	0.0077	21	**: (Inferior|Tongue base|No Margins . assessment in conjunction with separate|Medial|Deep|Lateral) margin**	Mixed concepts: SITE, TUMOUR LOCATION, SURGICAL CHARACTERISTICS	(S3c.93)
1.79	0.0077	21	**9 (:|.) .? Left**		(S3c.94)
1.43	0.0082	29	**three (lymph|possible|.lymph) nodes ;**		(S3c.95)
1.70	0.0083	20	**10 : Labelled . left? neck? (dissection|right)? level**		(S3c.96)
1.70	0.0083	20	**mm (in|at the) maximum**		(S3c.97)
2.76	0.0093	9	**7 (:|x 5 x) Labelled . right? (neck dissection|Right)? level? 3**		(S3c.98)
2.76	0.0093	9	**HPV studies P16 immunohistochemistry : (positive|Positive) In situ hybridisation**	HPV STATUS	(S3c.99)
2.76	0.0093	9	**No evidence of malignancy in eight lymph nodes? . 0.8**		(S3c.100)
2.76	0.0093	9	**cell carcinoma in one of? (three|two) of? (seven|five)? lymph nodes .? .**		(S3c.101)
2.76	0.0093	9	**cell carcinoma (,|is) HPV (studies to follow|related) .**	HPV STATUS	(S3c.102)
2.76	0.0093	9	**lymph node (,|. 0.1 . .|measuring 15 x)? 10**		(S3c.103)
2.76	0.0093	9	**2A- three lymph nodes ; 2B-? one? (largest|possible)? lymph? node**		(S3c.104)
2.76	0.0093	9	**: (Inferior|Medial|Lateral) margin .**	TUMOUR LOCATION	(S3c.105)
2.76	0.0093	9	**HPV (, and therefore|studies indicate) the**	HPV STATUS	(S3c.106)
2.76	0.0093	9	**, (the specimen consists of|39) (a|an orientated 58)? x? 28**		(S3c.107)

Log (OR)	P	N	Pattern	Comments	Crossref.
2.76	0.0093	9	**: Positive In situ hybridisation? .**		(S3c.108)
2.76	0.0093	9	**tonsil tumour? . ,**	SITE	(S3c.109)
2.76	0.0093	9	**right lateral? tongue base**	SITE	(S3c.110)
2.76	0.0093	9	**measures (30|20|45|approximately 11 x 10 x 5) mm**		(S3c.111)
2.76	0.0093	9	**margin .? (: No evidence|a clearance|being) of? malignancy .? 3**		(S3c.112)
2.76	0.0093	9	**is (an infiltrate of non-keratinized|extensive|metastatic) squamous cell carcinoma**		(S3c.113)
2.76	0.0093	9	**base (: Reactive lymphoid hyperplasia|tumour|.|margin) .**	SITE (Type IIIB MD)	(S3c.114)
2.76	0.0093	9	**appears (involved|unremarkable|normal) .**		(S3c.115)
2.76	0.0093	9	**and (squamous cell carcinoma|6C- one lymph node bisected|tonsil T1 : Tumour 2 cm or less|fibrous clefts) in**	SITE, STAGE	(S3c.116)
2.76	0.0093	9	**. (R|right) oropharyngectomy**		(S3c.117)
2.76	0.0093	9	**Description : Specimen 1 :? Labelled .? (Right|right)? lateral? tongue base**	SITE, TUMOUR LOCATION	(S3c.118)
2.76	0.0093	9	**: Labelled . right (2A|tonsil|oropharyngectomy) . , the specimen consists**	SITE	(S3c.119)
2.76	0.0093	9	**: Labelled . right (2A|oropharyngectomy|tongue base) . , the specimen**	SITE	(S3c.120)
2.76	0.0093	9	**3 : .? Right level? 2B? neck dissection? level 2B? (5A|.)? .**	NODAL	(S3c.121)
2.76	0.0093	9	**2 : Labelled .? (Inferior|deep|inferior|Deep) margin**	SURGICAL CHARACTERISTICS	(S3c.122)
2.76	0.0093	9	**Positive In situ hybridization for? (high risk|High)? (HPV genotypes|Comment|Maxmum size) :**	HPV STATUS	(S3c.123)
2.76	0.0093	9	**Right (neck dissection 1|2A|oropharyngectomy) .**		(S3c.124)
2.76	0.0093	9	**Right (2A|and left neck dissection|oropharyngectomy) :**		(S3c.125)
2.76	0.0093	9	**The (HPV studies|results) indicate**		(S3c.126)
2.76	0.0093	9	**The (largest|smaller involved)? lymph node**		(S3c.127)
2.76	0.0093	9	**. (right|left) level 1B**	NODAL	(S3c.128)
1.31	0.0093	44	**is (HPV related|5 mm|tumour free|positive|a likely primary site|present|seen macroscopically|serially sectioned|normal|clear of all margins|found|lymphoid hyperplasia with reactive germinal centers|included|posterolateral|uncertain) .**	Mixed concept: HPV STATUS, PATHOLOGY & SURGICAL CHARACTERISTICS	(S3c.129)
1.95	0.01	17	**in one of? (three|two) of? (seven|five)? lymph nodes .? .**		(S3c.130)
1.95	0.01	17	**Labelled . (tongue base|lateral|inferior|anterior|medial|superior) margin**	SITE, SURGICAL CHARACTERISTICS	(S3c.131)

Table S3: Full list of features listed mined by TEPAPA (Pathology reports) – (Cont'd)

Table S3(d): Positive binary features (regular expressions) with sequence-level annotation using UMLS vocabulary

Log (OR)	P	N	Pattern	Comments	Crossref.
3.50	$3{\times}10^{-6}$	25	**HPV (studies|genotypes|status) :? P16 immunohistochemistry :? Positive**	HPV STATUS	(S3d.1)
3.89	$6.2{\times}10^{-6}$	20	**for (high risk|High)? (HPV genotypes|P16) : Positive**	HPV STATUS	(S3d.2)
3.80	$1.6{\times}10^{-5}$	19	**tongue base (tumour|biopsy)? .**	SITE	(S3d.3)
3.80	$1.6{\times}10^{-5}$	19	**tongue base (tumour|biopsy)? .? ,**	SITE	(S3d.4)
3.80	$1.6{\times}10^{-5}$	19	**HPV (positive|genotypes : Positive|related) .**	HPV STATUS	(S3d.5)
3.23	$2.5{\times}10^{-5}$	22	**P16 immunohistochemistry? : Positive**	HPV STATUS	(S3d.6)
3.23	$2.5{\times}10^{-5}$	22	**. (Right|Left|right|left|right lateral)? tongue base**	SITE	(S3d.7)
2.68	$4.5{\times}10^{-5}$	25	**: (SCC R tonsil|1 and 2|Left inferior turbinate|Positive|One benign lymph node|Level 4|Level 5|See recent path|Reactive lymphoid hyperplasia|No malignancy|Not identified) .**	NODAL, SITE, HPV STATUS	(S3d.8)
3.53	$9.3{\times}10^{-5}$	16	**tongue base (tumour|.)? .**	SITE	(S3d.9)
3.53	$9.3{\times}10^{-5}$	16	**tongue base (tumour|biopsy)? . , the**	SITE	(S3d.10)
2.19	0.00011	30	**to (poorly differentiated squamous cell carcinoma . 2.9 .|lateral|deep|inferior|this|follow|squamous cell carcinoma in situ|2D|1C|3G) .**	TUMOUR LOCATION	(S3d.11)
3.05	0.00012	20	**Labelled . (right|left|right lateral)? tongue base**	SITE	(S3d.12)
3.43	0.00021	15	**high risk (. is positive|HPV genotypes : Positive) .**	HPV STATUS	(S3d.13)
2.96	0.00028	19	**of (HPV studies P16 immunohistochemistry : Positive In situ hybridisation|tumour|the specimen|all eight bocks|which show metastatic moderate to poorly differentiated squamous cell carcinoma|margins|human papilloma virus|basaloid type) .**	Mixed concepts: HPV STATUS, SURGICAL CHARACTERISTICS	(S3d.14)
2.96	0.00028	19	**: Labelled . (right|left|right lateral)? tongue base**	SITE	(S3d.15)
2.87	0.00062	18	**, (3|6 ,|the specimen consists of a piece of firm|deep margin inked black|skeletal muscle|minor salivary glands|keratinising|mucoserous glands) and**	PATHOLOGY FEATURE, SURGICAL CHARACTERISTICS	(S3d.16)
2.33	0.00064	21	**Labelled . (right|left|right lateral)? tongue**	SITE	(S3d.17)
1.83	0.00081	30	**of (HPV studies|seven lymph nodes|specimen 1|salivary gland|submandibular gland|tissue|all margins|this finding is uncertain|bone|non-keratinized squamous cell carcinoma|squamous cell carcinoma in situ|keratinization|fat) .**		(S3d.18)
3.23	0.001	13	**right (2A|tonsil|oropharyngectomy|tongue base) . , the specimen consists of**	SITE	(S3d.19)
3.23	0.001	13	**level (3|2A|5A|5B neck dissection|Vb) 9**		(S3d.20)
3.23	0.001	13	**: (SCC|Right|Left|Right lateral) tongue base**	SITE	(S3d.21)
3.13	0.0012	12	**HPV (studies indicate the|associated) squamous cell carcinoma**	HPV STATUS	(S3d.22)
3.13	0.0012	12	**level (1B|3|5A neck dissection|IIa) 8**		(S3d.23)
3.13	0.0012	12	**The (HPV studies|results)? indicate the?**	HPV STATUS	(S3d.24)

<table>
<tr><th>Log (OR)</th><th>P</th><th>N</th><th>Pattern</th><th>Comments</th><th>Crossref.</th></tr>
<tr><td></td><td></td><td></td><td>(tumour|squamous cell carcinoma) is positive for? HPV</td><td></td><td></td></tr>
<tr><td>3.13</td><td>0.0012</td><td>12</td><td>: The? (results|Metastatic)? HPV? studies? indicate the? (tumour|squamous cell carcinoma)? is HPV? related</td><td>HPV STATUS</td><td>(S3d.25)</td></tr>
<tr><td>3.13</td><td>0.0012</td><td>12</td><td>, (6|9 o.clock is black|poorly differentiated|non -keratinising|16|bone|tonsillar lymphoid tissue| therefore) ,</td><td>PATHOLOGY FEATURE, SITE</td><td>(S3d.26)</td></tr>
<tr><td>3.13</td><td>0.0012</td><td>12</td><td>Clinical Information : SCC? R</td><td></td><td>(S3d.27)</td></tr>
<tr><td>3.13</td><td>0.0012</td><td>12</td><td>mm (. macroscopic .|long|oriented mucosal excision with two sutures denoting anterior|tan| thick) and</td><td>TUMOUR LOCATION</td><td>(S3d.28)</td></tr>
<tr><td>3.13</td><td>0.0012</td><td>12</td><td>tumour (.|present) ,</td><td></td><td>(S3d.29)</td></tr>
<tr><td>2.23</td><td>0.0013</td><td>20</td><td>: Labelled . (right|left|right lateral)? tongue</td><td>SITE</td><td>(S3d.30)</td></tr>
<tr><td>2.67</td><td>0.0015</td><td>16</td><td>The (HPV studies|results)? (tumour|indicate) (appears to extend to|invades into)? the</td><td></td><td>(S3d.31)</td></tr>
<tr><td>3.01</td><td>0.0024</td><td>11</td><td>tongue base .? :</td><td>SITE</td><td>(S3d.32)</td></tr>
<tr><td>3.01</td><td>0.0024</td><td>11</td><td>the (tumour|squamous cell carcinoma) is HPV related</td><td>HPV STATUS</td><td>(S3d.33)</td></tr>
<tr><td>3.01</td><td>0.0024</td><td>11</td><td>squamous cell carcinoma (,|is) positive for? HPV</td><td>HPV STATUS</td><td>(S3d.34)</td></tr>
<tr><td>3.01</td><td>0.0024</td><td>11</td><td>Right (tonsil|oropharyngectomy)? (2A|tumour)? .</td><td>SITE</td><td>(S3d.35)</td></tr>
<tr><td>3.01</td><td>0.0024</td><td>11</td><td>. (right|left) neck? level 5A</td><td>NODAL</td><td>(S3d.36)</td></tr>
<tr><td>3.01</td><td>0.0024</td><td>11</td><td>P16 (is|immunohistochemistry : Positive|are both|+ve) positive? .</td><td>HPV STATUS</td><td>(S3d.37)</td></tr>
<tr><td>3.01</td><td>0.0024</td><td>11</td><td>: The? (HPV studies|results|Positivity for P53 on immunohistochemistry may) indicate</td><td></td><td>(S3d.38)</td></tr>
<tr><td>3.01</td><td>0.0024</td><td>11</td><td>: (Metastatic squamous cell carcinoma|Positive In situ hybridisation|Moderately differentiated) . (Ventana INFORM ISH|1.9|Grade 2 of 3) .</td><td></td><td>(S3d.39)</td></tr>
<tr><td>3.01</td><td>0.0024</td><td>11</td><td>: (The|Metastatic)? HPV (associated|related)? (studies indicate the|moderately differentiated| moderate to poorly differentiated)? squamous cell carcinoma</td><td>HPV STATUS</td><td>(S3d.40)</td></tr>
<tr><td>3.01</td><td>0.0024</td><td>11</td><td>: P16 immunohistochemistry :? Positive (In situ hybridisation . Ventana INFORM ISH .|In situ hybridization) for</td><td>HPV STATUS</td><td>(S3d.41)</td></tr>
<tr><td>3.01</td><td>0.0024</td><td>11</td><td>: The (HPV studies|results)? indicate the? (tumour|squamous cell carcinoma) is positive for? HPV</td><td>HPV STATUS</td><td>(S3d.42)</td></tr>
<tr><td>3.01</td><td>0.0024</td><td>11</td><td>, (HPV studies|3|two of which show metastatic moderate|invading) to</td><td></td><td>(S3d.43)</td></tr>
<tr><td>2.57</td><td>0.0029</td><td>15</td><td>seven (lymph nodes|nodes) (.|identified are all negative for malignancy)? .</td><td></td><td>(S3d.44)</td></tr>
<tr><td>2.57</td><td>0.0029</td><td>15</td><td>. (Right|Left|right|left|right lateral) tongue</td><td>SITE</td><td>(S3d.45)</td></tr>
<tr><td>2.57</td><td>0.0029</td><td>15</td><td>10 nodes? . .</td><td></td><td>(S3d.46)</td></tr>
<tr><td>1.88</td><td>0.0038</td><td>22</td><td>the (posterior half|centre)? (of the|multiple levels examined .|entire) specimen</td><td></td><td>(S3d.47)</td></tr>
<tr><td>2.89</td><td>0.0047</td><td>10</td><td>: (Metastatic|Squamous cell carcinoma .)? HPV related moderate to? (poorly|Grade 3 .poorly) differentiated</td><td>HPV STATUS</td><td>(S3d.48)</td></tr>
<tr><td>2.89</td><td>0.0047</td><td>10</td><td>5C- (four|five)? possible? lymph nodes ; 5D-?</td><td></td><td>(S3d.49)</td></tr>
</table>

Log (OR)	P	N	Pattern	Comments	Crossref.
			two? possible? lymph nodes		
2.89	0.0047	10	the (inferior|other)? (deep|half|false) margin? is inked black	SURGICAL CHARACTERISTICS	(S3d.50)
2.89	0.0047	10	right (neck level 4|tonsil|oropharyngectomy)? . , the specimen consists of a	NODAL, SITE	(S3d.51)
2.89	0.0047	10	lymph nodes (.|identified are all negative for malignancy)? . (0.|0.8|0.3) .? .? 10		(S3d.52)
2.89	0.0047	10	a large single lymph node exhibiting metastatic cystic? moderately differentiated non -keratinising? squamous cell carcinoma	PATHOLOGY FEATURE	(S3d.53)
2.89	0.0047	10	2A (neck dissection|.|+ 3) :		(S3d.54)
2.89	0.0047	10	10 :? (.|Labelled)? .? (Left|left) neck		(S3d.55)
2.89	0.0047	10	. (right|left) neck? level 5B	NODAL	(S3d.56)
2.89	0.0047	10	The (HPV studies|results)? (indicate|roughened surface is inked blue and) the (tumour|specimen|squamous cell carcinoma) is		(S3d.57)
2.89	0.0047	10	ISH .? for high risk HPV genotypes : Positive	HPV STATUS	(S3d.58)
2.89	0.0047	10	in the (posterior half|centre)? (of the|multiple levels examined .) specimen		(S3d.59)
2.89	0.0047	10	: (SCC R|Right|Labelled .) biopsy? right? tonsil	SITE	(S3d.60)
2.89	0.0047	10	: (1 Laterality|Squamous cell carcinoma|Results|Moderately differentiated . Grade 2|Approximately 50%) of		(S3d.61)
2.89	0.0047	10	, (poorly differentiated|non -keratinising) squamous cell carcinoma	PATHOLOGY FEATURE	(S3d.62)
2.89	0.0047	10	indicate the (tumour|squamous cell carcinoma) is HPV related	HPV STATUS	(S3d.63)
2.89	0.0047	10	pieces of (red|tan)? (fatty tissue|tissue) ,		(S3d.64)
2.89	0.0047	10	2 : Right neck dissection? (level 1B|tonsil|oropharyngectomy) .	NODAL, SITE	(S3d.65)
2.89	0.0047	10	30 x 20 x? 10 mm in aggregate? .		(S3d.66)
2.89	0.0047	10	4 : Labelled . right? neck dissection? (level 4|left level IIa) . ,	NODAL	(S3d.67)
2.89	0.0047	10	COMMENT : The? (HPV studies|results|Positivity for P53 on immunohistochemistry may) indicate		(S3d.68)
2.89	0.0047	10	P16 immunohistochemistry : (positive|Positive) In situ hybridisation	HPV STATUS	(S3d.69)
2.89	0.0047	10	Block 4A- (three|two|six possible) .? lymph nodes		(S3d.70)
2.04	0.0051	18	. (tongue base|lateral|inferior|anterior|medial|superior|Negative|Deep) margin	SITE, HPV STATUS, TUMOUR LOCATION, SURGICAL CHARACTERISTICS	(S3d.71)
2.04	0.0051	18	the (specimen consists of a piece of firm|margin is inked blue|anterior|jugular vein|first cystic cavity ; 8E-) and	Mixed concepts: TUMOUR LOCATION, SURGICAL CHARACTERISTICS	(S3d.72)
2.04	0.0051	18	tumour (is present at|. ,|appears to extend to|invades into) the		(S3d.73)
1.42	0.0053	35	and (2|1B|2B|3B|both show cystic degeneration|	Mixed concepts:	(S3d.74)

<table>
<tr><th>Log (OR)</th><th>P</th><th>N</th><th>Pattern</th><th>Comments</th><th>Crossref.</th></tr>
<tr><td></td><td></td><td></td><td>all embedded Block 1A|anterior|skeletal muscle|focal acute on chronic inflammation identified|bone|contains brown watery fluid| minor salivary glands|9D .|squamous cell carcinoma in situ|neutrophils|tonsillar lymphoid tissue|1H - epiglottis transverse sections| mucoserous glands|skeletal muscles) .</td><td>PATHOLOGY FEATURE, SITE, TUMOUR LOCATION</td><td></td></tr>
<tr><td>2.47</td><td>0.0057</td><td>14</td><td>Right (level 4|2A|oropharyngectomy|tongue base) :</td><td>NODAL, SITE</td><td>(S3d.75)</td></tr>
<tr><td>2.47</td><td>0.0057</td><td>14</td><td>studies :? P16 immunohistochemistry : Positive</td><td>HPV STATUS</td><td>(S3d.76)</td></tr>
<tr><td>2.47</td><td>0.0057</td><td>14</td><td>: (SCC|Right|Left|Right lateral) tongue</td><td>SITE</td><td>(S3d.77)</td></tr>
<tr><td>2.47</td><td>0.0057</td><td>14</td><td>: The? (HPV studies|results)? (tumour invades into|indicate|Approximately 50% of) the</td><td></td><td>(S3d.78)</td></tr>
<tr><td>1.56</td><td>0.0065</td><td>27</td><td>of (three|seven|four|five|eight|six|thirteen| seventeen|twenty one) lymph nodes</td><td></td><td>(S3d.79)</td></tr>
<tr><td>1.79</td><td>0.0077</td><td>21</td><td>1B (3|6|8|.) .</td><td></td><td>(S3d.80)</td></tr>
<tr><td>1.79</td><td>0.0077</td><td>21</td><td>9 (:|.) .? Left</td><td></td><td>(S3d.81)</td></tr>
<tr><td>1.79</td><td>0.0077</td><td>21</td><td>: (Inferior|Tongue base|No Margins . assessment in conjunction with separate| Medial|Deep|Lateral) margin</td><td>SITE, TUMOUR LOCATION, SURGICAL CHARACTERISTICS</td><td>(S3d.82)</td></tr>
<tr><td>1.43</td><td>0.0082</td><td>29</td><td>three (possible nodes|.)? lymph nodes? ;</td><td></td><td>(S3d.83)</td></tr>
<tr><td>1.70</td><td>0.0083</td><td>20</td><td>right (level 4|2A|tonsil|oropharyngectomy| tongue base|level III) .</td><td>NODAL, SITE</td><td>(S3d.84)</td></tr>
<tr><td>1.70</td><td>0.0083</td><td>20</td><td>mm (in|at the) maximum</td><td></td><td>(S3d.85)</td></tr>
<tr><td>2.76</td><td>0.0093</td><td>9</td><td>, (the specimen consists of|39) (a|an orientated 58)? x? 28</td><td></td><td>(S3d.86)</td></tr>
<tr><td>2.76</td><td>0.0093</td><td>9</td><td>. (R|right) oropharyngectomy</td><td>MANAGEMENT</td><td>(S3d.87)</td></tr>
<tr><td>2.76</td><td>0.0093</td><td>9</td><td>The (HPV studies|results) indicate</td><td></td><td>(S3d.88)</td></tr>
<tr><td>2.76</td><td>0.0093</td><td>9</td><td>: (No evidence of|No dysplasia or)? (1 and| malignancy|Tumour) 2</td><td>PATHOLOGY FEATURE</td><td>(S3d.89)</td></tr>
<tr><td>2.76</td><td>0.0093</td><td>9</td><td>a (tumour|fibrofatty tissue|fatty tissue|poorly differentiated|P53 mutation) ,</td><td>PATHOLOGY FEATURE</td><td>(S3d.90)</td></tr>
<tr><td>2.76</td><td>0.0093</td><td>9</td><td>appears (involved|unremarkable|normal) .</td><td></td><td>(S3d.91)</td></tr>
<tr><td>2.76</td><td>0.0093</td><td>9</td><td>margin .? (: No evidence of malignancy .|a clearance of|being) 3</td><td></td><td>(S3d.92)</td></tr>
<tr><td>2.76</td><td>0.0093</td><td>9</td><td>: (Inferior|Medial|Lateral) margin .</td><td>TUMOUR LOCATION</td><td>(S3d.93)</td></tr>
<tr><td>2.76</td><td>0.0093</td><td>9</td><td>squamous cell carcinoma (,|is) HPV (studies to follow|related) .</td><td>HPV STATUS</td><td>(S3d.96)</td></tr>
<tr><td>2.76</td><td>0.0093</td><td>9</td><td>HPV studies P16 immunohistochemistry : (positive|Positive) In situ hybridisation</td><td>HPV STATUS</td><td>(S3d.97)</td></tr>
<tr><td>2.76</td><td>0.0093</td><td>9</td><td>. (right|left) level 1B</td><td>NODAL</td><td>(S3d.98)</td></tr>
<tr><td>2.76</td><td>0.0093</td><td>9</td><td>HPV (, and therefore|studies indicate) the</td><td>HPV STATUS</td><td>(S3d.100)</td></tr>
<tr><td>2.76</td><td>0.0093</td><td>9</td><td>2 : Labelled . right neck dissection level? (1B| level 4|1b) .</td><td>NODAL</td><td>(S3d.101)</td></tr>
<tr><td>2.76</td><td>0.0093</td><td>9</td><td>Positive (In situ hybridisation . Ventana INFORM ISH .|In situ hybridization) for high risk</td><td>HPV STATUS</td><td>(S3d.102)</td></tr>
<tr><td>2.76</td><td>0.0093</td><td>9</td><td>Positive In situ hybridization for? (high risk| High)? (HPV genotypes|Comment|Maxmum size) :</td><td>HPV STATUS</td><td>(S3d.103)</td></tr>
<tr><td>2.76</td><td>0.0093</td><td>9</td><td>2 : Labelled .? (Inferior|deep|inferior|Deep)</td><td>SURGICAL</td><td>(S3d.104)</td></tr>
</table>

Log (OR)	P	N	Pattern	Comments	Crossref.
			**margin**	CHARACTERISTICS	
2.76	0.0093	9	**3 : .? Right level? 2B? neck dissection? level 2B? (5A|.)? .**	NODAL	(S3d.105)
2.76	0.0093	9	**4 : Right? neck dissection? (level 4|Left level IIa|Level 4) .**	NODAL	(S3d.106)
2.76	0.0093	9	**: Positive In situ hybridisation? .**		(S3d.107)
2.76	0.0093	9	**No evidence of malignancy in eight lymph nodes? . 0.8**		(S3d.109)
1.95	0.01	17	**Labelled . (tongue base|lateral|inferior|anterior|medial|superior) margin**	SITE, TUMOUR LOCATION, SURGICAL CHARACTERISTICS	(S3d.110)
1.95	0.01	17	**in one of? (three|two) of? (seven|five)? lymph nodes .? .**		(S3d.111)

Table S3: Full list of features listed mined by TEPAPA (Pathology reports) – (Cont'd)

Table S3(e): Negative binary features (n-gram) without sequence-level annotation

Log (OR)	P	N	Pattern	Comments	Crossref.
-2.37	0.00029	16	**: Negative**	HPV STATUS (Type IIIA MD)	(S3e.1)
-3.54	0.00035	8	**for decalcification**	TUMOUR LOCATION (Type IIIA MD)	(S3e.2)
-3.54	0.00035	8	**The sections of the**		(S3e.3)
-2.94	0.00081	10	**with mild**		(S3e.4)
-3.37	0.001	7	**Specimen 3 : " Left**		(S3e.5)
-3.37	0.001	7	**lobe of**		(S3e.6)
-3.37	0.001	7	**a depth of 3 mm**		(S3e.7)
-3.37	0.001	7	**: Sections**		(S3e.8)
-2.09	0.0018	14	**are clear**		(S3e.9)
-1.56	0.002	31	**This**		(S3e.10)
-2.77	0.0022	9	**o'clock margin**	SURGICAL CHARACTERISTICS	(S3e.11)
-2.77	0.0022	9	**posterior aspect**		(S3e.12)
-2.37	0.0023	12	**invasive malignancy .**		(S3e.13)
-3.18	0.0029	6	**the possibility of**		(S3e.14)
-3.18	0.0029	6	**soft tissue and**		(S3e.15)
-3.18	0.0029	6	**post**		(S3e.16)
-1.53	0.0034	29	**2 mm .**		(S3e.17)
-1.52	0.0044	45	**seen .**		(S3e.18)
-1.67	0.0049	17	**inflammation .**		(S3e.19)
-2.22	0.0053	11	**formation**		(S3e.20)
-2.22	0.0053	11	**mild chronic**		(S3e.21)
-2.59	0.0058	8	**5 x 5 x**		(S3e.22)
-2.59	0.0058	8	**differentiated , keratinising squamous cell carcinoma**	PATHOLOGY FEATURE	(S3e.23)
-2.59	0.0058	8	**involve**		(S3e.24)
-2.59	0.0058	8	**the soft**		(S3e.25)
-2.59	0.0058	8	**well differentiated**	PATHOLOGY FEATURE	(S3e.26)
-2.59	0.0058	8	**x 7 x 3 mm**		(S3e.27)
-1.78	0.0065	15	**focus**		(S3e.28)
-2.98	0.0083	5	**9 x 6**		(S3e.29)
-2.98	0.0083	5	**Block 2A- five**		(S3e.30)
-2.98	0.0083	5	**effect .**		(S3e.31)
-2.98	0.0083	5	**neural**		(S3e.32)
-2.98	0.0083	5	**suspicious for**		(S3e.33)
-1.34	0.0088	33	**area**		(S3e.34)
-1.31	0.0093	31	**features**		(S3e.35)
-1.33	0.0099	41	**show a**		(S3e.36)
-1.40	0.0099	22	**margins are**		(S3e.37)

Table S3: Full list of features listed mined by TEPAPA (Pathology reports) – (Cont'd)

Table S3(f): Negative binary features (n-gram) with sequence-level annotation using UMLS vocabulary

Log (OR)	P	N	Pattern	Comments	Crossref.
-2.37	0.00029	16	**: Negative**	HPV STATUS (Type IIIB MD)	(S3f.1)
-3.54	0.00035	8	**for decalcification**	PATHOLOGY FEATURE	(S3f.2)
-3.54	0.00035	8	**The sections of the**		(S3f.3)
-3.37	0.001	7	**a depth of 3 mm**		(S3f.4)
-3.37	0.001	7	**more**		(S3f.5)
-3.37	0.001	7	**: Sections**		(S3f.6)
-2.09	0.0018	14	**are clear**		(S3f.7)
-1.56	0.002	31	**This**		(S3f.8)
-2.77	0.0022	9	**posterior aspect**		(S3f.9)
-2.37	0.0023	12	**invasive malignancy .**		(S3f.10)
-1.74	0.0028	20	**invasion is**		(S3f.11)
-3.18	0.0029	6	**lobe of**		(S3f.12)
-3.18	0.0029	6	**the possibility of**		(S3f.13)
-3.18	0.0029	6	**soft tissue and**		(S3f.14)
-1.53	0.0034	29	**2 mm .**		(S3f.15)
-2.59	0.0058	8	**blue inked**		(S3f.16)
-2.59	0.0058	8	**lateral aspect of**		(S3f.17)
-2.59	0.0058	8	**well differentiated**	PATHOLOGY FEATURE	(S3f.18)
-2.59	0.0058	8	**, keratinising squamous cell carcinoma**	PATHOLOGY FEATURE	(S3f.19)
-2.59	0.0058	8	**5 x 5 x**		(S3f.20)
-2.59	0.0058	8	**involve**		(S3f.21)
-2.59	0.0058	8	**x 7 x 3 mm**		(S3f.22)
-1.78	0.0065	15	**focus**		(S3f.23)
-2.98	0.0083	5	**anterior commissure**		(S3f.24)
-2.98	0.0083	5	**, focal**		(S3f.25)
-2.98	0.0083	5	**with mild chronic inflammation**	PATHOLOGY FEATURE	(S3f.26)
-2.98	0.0083	5	**suspicious for**		(S3f.27)
-2.98	0.0083	5	**neural**		(S3f.28)
-2.98	0.0083	5	**effect .**		(S3f.29)
-2.98	0.0083	5	**Specimen 3 : " Left**		(S3f.30)
-2.98	0.0083	5	**and in**		(S3f.31)
-2.98	0.0083	5	**Block 2A - five**		(S3f.32)
-2.98	0.0083	5	**/2015 11 :**		(S3f.33)
-2.98	0.0083	5	**but the**		(S3f.34)
-2.98	0.0083	5	**9 x 6**		(S3f.35)
-1.34	0.0088	33	**area**		(S3f.36)
-1.31	0.0093	31	**features**		(S3f.37)
-1.33	0.0099	41	**Negative**	HPV STATUS (Type IIIB MD)	(S3f.38)
-1.33	0.0099	41	**show a**		(S3f.39)

Table S3: Full list of features listed mined by TEPAPA (Pathology reports) – (Cont'd)

Table S3(g): Negative binary features (regular expressions) without sequence-level annotation

<table>
<tr><th>Log (OR)</th><th>P</th><th>N</th><th>Pattern</th><th>Comments</th><th>Crossref.</th></tr>
<tr><td>-3.24</td><td>9.7×10^{-5}</td><td>12</td><td>The paraffin? sections of</td><td></td><td>(S3g.1)</td></tr>
<tr><td>-3.69</td><td>0.00011</td><td>9</td><td>consists of (three|a|one|two)? 3 mm</td><td></td><td>(S3g.2)</td></tr>
<tr><td>-2.21</td><td>0.00014</td><td>21</td><td>The (sections|base|central portion|lesion is 2 mm clear|cut surface|edge|remainder| outside) of the</td><td>SITE (Type IIIB MD)</td><td>(S3g.3)</td></tr>
<tr><td>-2.66</td><td>0.00016</td><td>14</td><td>for (high risk HPV genotypes : Negative| decalcification) prior to further dissection? .</td><td>HPV STATUS, TUMOUR LOCATION (Type IIIB MD)</td><td>(S3g.4)</td></tr>
<tr><td>-2.66</td><td>0.00016</td><td>14</td><td>of metastatic moderate to? (poorly| moderately|well) differentiated</td><td>PERFORMANCE STATUS</td><td>(S3g.5)</td></tr>
<tr><td>-3.09</td><td>0.00029</td><td>11</td><td>, (invasive|moderately differentiated| keratinising) squamous cell carcinoma</td><td>PATHOLOGY FEATURE</td><td>(S3g.6)</td></tr>
<tr><td>-3.54</td><td>0.00035</td><td>8</td><td>medial tongue? (. ,|aspect of) the</td><td>SITE</td><td>(S3g.7)</td></tr>
<tr><td>-3.54</td><td>0.00035</td><td>8</td><td>. , the specimen consists of (9 x|three|one| two)? 3</td><td></td><td>(S3g.8)</td></tr>
<tr><td>-2.52</td><td>0.00046</td><td>13</td><td>mm (mucosal|pale|tan|punch) biopsy</td><td>TUMOUR LOCATION (Type IIIB MD)</td><td>(S3g.9)</td></tr>
<tr><td>-2.06</td><td>0.00071</td><td>17</td><td>the (lateral|inferior|medial|posterior) aspect</td><td>TUMOUR LOCATION (Type IIIB MD)</td><td>(S3g.10)</td></tr>
<tr><td>-3.37</td><td>0.001</td><td>7</td><td>with (high|low)? (grade|mild) dysplasia</td><td>PATHOLOGY FEATURE</td><td>(S3g.11)</td></tr>
<tr><td>-3.37</td><td>0.001</td><td>7</td><td>right (neck|temple) dissection? :</td><td></td><td>(S3g.12)</td></tr>
<tr><td>-3.37</td><td>0.001</td><td>7</td><td>of (moderately|metastatic moderate to poorly) differentiated , keratinising? squamous cell</td><td>PATHOLOGY FEATURE</td><td>(S3g.13)</td></tr>
<tr><td>-3.37</td><td>0.001</td><td>7</td><td>with mild (dysplasia|chronic inflammation) .</td><td>PATHOLOGY FEATURE</td><td>(S3g.14)</td></tr>
<tr><td>-1.65</td><td>0.0014</td><td>26</td><td>the (level|base|larger piece|surface|region| margins|majority|edge|edges|proximal margin|request|shape|possibility|rest| periphery) of</td><td>SITE</td><td>(S3g.15)</td></tr>
<tr><td>-1.64</td><td>0.0019</td><td>35</td><td>the (squamous epithelium|carcinoma|tumour| right|largest bisected|surface|maximum dimension|bone|other end|margins|lesion| report is missing|biopsy|multiple sections examined|stroma|lip|floor of mouth|thyroid cartilage|following features|diagnosis| opposite side) .</td><td></td><td>(S3g.16)</td></tr>
<tr><td>-1.81</td><td>0.0021</td><td>18</td><td>. (for high risk HPV genotypes : Negative| lymph nodes|.|dysplasia|margins|Alarm continuation sequencing error) .</td><td>HPV STATUS, PATHOLOGY FEATURE</td><td>(S3g.17)</td></tr>
<tr><td>-2.77</td><td>0.0022</td><td>9</td><td>12 o.clock superior? (. ,|and|margin of) the</td><td></td><td>(S3g.18)</td></tr>
<tr><td>-2.77</td><td>0.0022</td><td>9</td><td>sections of (the|salivary)? submandibular? (3 and 9 o.clock|the|gland) (are unremarkable| ends)? respectively? .</td><td></td><td>(S3g.19)</td></tr>
<tr><td>-2.77</td><td>0.0022</td><td>9</td><td>: (P16 immunohistochemistry :|Squamous cell carcinoma)? (An|Negative In)? in? situ</td><td>HPV STATUS</td><td>(S3g.20)</td></tr>
<tr><td>-2.77</td><td>0.0022</td><td>9</td><td>differentiated squamous cell? (carcinoma| adenocarcinoma) with</td><td></td><td>(S3g.21)</td></tr>
</table>

<table>
<tr><th>Log (OR)</th><th>P</th><th>N</th><th>Pattern</th><th>Comments</th><th>Crossref.</th></tr>
<tr><td>-2.37</td><td>0.0023</td><td>12</td><td>consists of (three|a|one|two)? 3</td><td></td><td>(S3g.22)</td></tr>
<tr><td>-3.18</td><td>0.0029</td><td>6</td><td>of (the|resection are clear) (right side|lesion appears complete and)? of? the</td><td></td><td>(S3g.23)</td></tr>
<tr><td>-3.18</td><td>0.0029</td><td>6</td><td>tissue (: No evidence of malignancy .|,) 6</td><td></td><td>(S3g.24)</td></tr>
<tr><td>-3.18</td><td>0.0029</td><td>6</td><td>the (carcinoma with|specimen show|lesion appears complete and) the</td><td></td><td>(S3g.25)</td></tr>
<tr><td>-3.18</td><td>0.0029</td><td>6</td><td>studies :? (for high risk HPV|P16 immunohistochemistry : Negative In situ hybridisation) .</td><td>HPV STATUS</td><td>(S3g.26)</td></tr>
<tr><td>-3.18</td><td>0.0029</td><td>6</td><td>of (tumour|the submandibular gland| resection) are</td><td></td><td>(S3g.27)</td></tr>
<tr><td>-3.18</td><td>0.0029</td><td>6</td><td>of one punch biopsy ,? 2 x 2? mm .</td><td>TUMOUR LOCATION (Type IIIB MD)</td><td>(S3g.28)</td></tr>
<tr><td>-3.18</td><td>0.0029</td><td>6</td><td>medial tongue? . , the specimen</td><td>SITE</td><td>(S3g.29)</td></tr>
<tr><td>-3.18</td><td>0.0029</td><td>6</td><td>biopsy of skin? (to|shows) a</td><td></td><td>(S3g.30)</td></tr>
<tr><td>-3.18</td><td>0.0029</td><td>6</td><td>a (small|large) area</td><td></td><td>(S3g.31)</td></tr>
<tr><td>-3.18</td><td>0.0029</td><td>6</td><td>4 (:|.) . Left</td><td></td><td>(S3g.32)</td></tr>
<tr><td>-3.18</td><td>0.0029</td><td>6</td><td>Block 2A- (four possible|five) lymph? nodes ; 2B-</td><td></td><td>(S3g.33)</td></tr>
<tr><td>-3.18</td><td>0.0029</td><td>6</td><td>1 : (.|Deep medial margin)? Right? (level IIb| vocal cord .)? .</td><td>NODAL, SITE</td><td>(S3g.34)</td></tr>
<tr><td>-1.92</td><td>0.003</td><td>16</td><td>of (three|one|two)? 3 mm</td><td></td><td>(S3g.35)</td></tr>
<tr><td>-1.53</td><td>0.0034</td><td>29</td><td>sections (of|; 3E-|from) the</td><td></td><td>(S3g.36)</td></tr>
<tr><td>-1.49</td><td>0.0042</td><td>34</td><td>2 mm (of extranodal spread|pale biopsy|from the deep margin|fragments|biopsies)? .</td><td>PATHOLOGY FEATURE</td><td>(S3g.37)</td></tr>
<tr><td>-1.56</td><td>0.0044</td><td>21</td><td>is (bisected|black|focal mild dysplasia|noted| pending|consistent with a recurrence| difficult|required|missing|1.2 mm| recommended) .</td><td>PATHOLOGY FEATURE</td><td>(S3g.38)</td></tr>
<tr><td>-1.56</td><td>0.0044</td><td>21</td><td>the (right|black inked|medial|surgical|distal tracheal|proximal) margin</td><td></td><td>(S3g.39)</td></tr>
<tr><td>-1.95</td><td>0.0044</td><td>13</td><td>of (three|the|one|two|malignancy . 0.2 . .| slice) 3</td><td></td><td>(S3g.40)</td></tr>
<tr><td>-1.95</td><td>0.0044</td><td>13</td><td>of (moderately|metastatic) moderate to poorly? differentiated? , keratinising? squamous cell carcinoma</td><td>PATHOLOGY FEATURE</td><td>(S3g.41)</td></tr>
<tr><td>-1.95</td><td>0.0044</td><td>13</td><td>HPV (studies|genotypes) :? P16 immunohistochemistry :? Negative</td><td>HPV STATUS</td><td>(S3g.42)</td></tr>
<tr><td>-1.67</td><td>0.0049</td><td>17</td><td>mm (.|right|deep|anterior|medial|superior| thick , extending|punch biopsy) to</td><td>TUMOUR LOCATION (Type IIIB MD)</td><td>(S3g.43)</td></tr>
<tr><td>-2.22</td><td>0.0053</td><td>11</td><td>Labelled . (medial tongue|posterior margin| total laryngectomy|larynx|subtotal glossectomy) . , the specimen consists of</td><td>SITE</td><td>(S3g.44)</td></tr>
<tr><td>-2.22</td><td>0.0053</td><td>11</td><td>Labelled . (right vocal cord|medial tongue| total laryngectomy|larynx|subtotal glossectomy) . , the</td><td>SITE</td><td>(S3g.45)</td></tr>
<tr><td>-2.22</td><td>0.0053</td><td>11</td><td>There is no evidence of? (in situ|high grade dysplasia)? or? invasive</td><td>PATHOLOGY FEATURE</td><td>(S3g.46)</td></tr>
<tr><td>-2.22</td><td>0.0053</td><td>11</td><td>of skin to? a (6 x|depth of)? 3</td><td></td><td>(S3g.47)</td></tr>
<tr><td>-2.59</td><td>0.0058</td><td>8</td><td>soft tissue margin? ;</td><td></td><td>(S3g.48)</td></tr>
<tr><td>-2.59</td><td>0.0058</td><td>8</td><td>evidence of high grade dysplasia or? invasive malignancy .</td><td>PATHOLOGY FEATURE</td><td>(S3g.49)</td></tr>
</table>

Log (OR)	P	N	Pattern	Comments	Crossref.
-2.59	0.0058	8	**3 :? (.|Labelled)? . Left**		(S3g.50)
-2.59	0.0058	8	**SUMMARY 1 .? (:|.) Left**		(S3g.51)
-2.59	0.0058	8	**a (6 x|depth of) 3**		(S3g.52)
-2.59	0.0058	8	**no (invasive malignancy|dysplasia|perineural invasion|significant change) .**	PATHOLOGY FEATURE	(S3g.53)
-2.59	0.0058	8	**of (submandibular gland|resection are clear of)? (the lesion|margins)? ;**		(S3g.54)
-2.59	0.0058	8	**one (end|transverse section|edge) of the**		(S3g.55)
-1.60	0.006	19	**The (base|excision|central portion|focus|regions|presence|edge|remainder|outside) of**	SITE (Type IIIB MD)	(S3g.56)
-1.78	0.0065	15	**high grade dysplasia? (risk HPV genotypes : Negative|or invasive malignancy)? .**	HPV STATUS, PATHOLOGY FEATURE	(S3g.57)
-2.98	0.0083	5	**the closest (transverse|peripheral)? margin**		(S3g.58)
-2.98	0.0083	5	**: Labelled . (deep medial|posterior) margin . , the specimen**	TUMOUR LOCATION (Type IIIB MD)	(S3g.59)
-2.98	0.0083	5	**HPV studies :? P16 immunohistochemistry : Negative In situ**	HPV STATUS	(S3g.60)
-2.98	0.0083	5	**are clear (of|from) the**		(S3g.61)
-2.98	0.0083	5	**consists of (a|two pale tan biopsies ,) 4**		(S3g.62)
-2.98	0.0083	5	**punch biopsy of skin? to a depth**	TUMOUR LOCATION (Type IIIB MD)	(S3g.63)
-2.98	0.0083	5	**with (high|low) grade**		(S3g.64)
-2.98	0.0083	5	**well differentiated squamous cell carcinoma? ,**	PATHOLOGY FEATURE	(S3g.65)
-2.98	0.0083	5	**3 (mm|o.clock) to**		(S3g.66)
-2.98	0.0083	5	**. lesion? (right|left) posterior**	TUMOUR LOCATION (Type IIIB MD)	(S3g.67)
-2.98	0.0083	5	**and the? underlying skeletal**		(S3g.68)
-2.98	0.0083	5	**are (clear from the|biopsies of moderately differentiated , keratinising squamous cell) carcinoma**	PATHOLOGY FEATURE	(S3g.69)
-2.98	0.0083	5	**Both (ends|negative) .**		(S3g.70)
-2.98	0.0083	5	**of skin to? a depth of? 4**		(S3g.71)
-2.98	0.0083	5	**the (submandibular gland|appearances) are**		(S3g.72)
-1.40	0.0099	22	**differentiated ,? (invasive|keratinizing|keratinising) squamous cell carcinoma**	PATHOLOGY FEATURE	(S3g.73)

Table S3: Full list of features listed mined by TEPAPA (Pathology reports) – (Cont'd)

Table S3(h): Negative binary features (regular expressions) with sequence-level annotation using UMLS vocabulary

Log (OR)	P	N	Pattern	Comments	Crossref.
-3.98	1.1×10^{-5}	11	**of (two pale tan biopsies ,|slice|skin to)? a? depth of? 4**		(S3h.1)
-3.24	9.7×10^{-5}	12	**The paraffin? sections of**		(S3h.2)
-3.69	0.00011	9	**consists of (three|a|one|two)? 3 mm**		(S3h.3)
-2.08	0.00012	39	**the (tumour|right|largest bisected|salivary gland|surface|squamous epithelium|carcinoma|maximum dimension|bone|margins|nodes|lesion|report is missing|biopsy|multiple sections examined|stroma|lip|surgical margin|floor of mouth|thyroid cartilage|following features|other end|diagnosis|opposite side) .**		(S3h.4)
-2.21	0.00014	21	**The (sections|base|central portion|lesion is 2 mm clear|cut surface|edge|remainder|outside) of the**	SITE (Type IIIB MD)	(S3h.5)
-2.66	0.00016	14	**for (high risk HPV genotypes : Negative|decalcification) prior to further dissection? .**	HPV STATUS, TUMOUR LOCATION (Type IIIB MD)	(S3h.6)
-3.09	0.00029	11	**, (invasive|moderately differentiated|keratinising) squamous cell carcinoma**	PATHOLOGY FEATURE	(S3h.7)
-3.54	0.00035	8	**. , the specimen consists of (9 x|three|one|two)? 3**		(S3h.8)
-3.54	0.00035	8	**medial tongue? (. ,|aspect of) the**	SITE	(S3h.9)
-1.94	0.00039	26	**the (level|right half|base|larger piece|surface|region|margins|majority|right side|edge|edges|proximal margin|request|shape|possibility|rest|periphery) of**	SITE (Type IIIB MD)	(S3h.10)
-2.06	0.00071	17	**the (lateral|inferior|medial|posterior) aspect**	TUMOUR LOCATION (Type IIIB MD)	(S3h.11)
-2.23	0.00074	15	**with (squamous cell carcinoma|minor salivary gland|cyst formation|previous surgery|mild chronic inflammation|mild dysplasia|prominent nucleoli) .**	PATHOLOGY FEATURE	(S3h.12)
-1.66	0.0014	28	**sections (of|from) the**		(S3h.13)
-2.09	0.0018	14	**of (tumour|one punch biopsy|two pale tan biopsies|soft tissue|moderately differentiated|multiple unoriented pieces of fibrofatty tissue|nose|mucosal tissue) ,**	TUMOUR LOCATION (Type IIIA MD)	(S3h.14)
-1.81	0.0021	18	**. (lymph nodes|.|for high risk HPV genotypes : Negative|dysplasia|margins|Alarm continuation sequencing error) .**	HPV STATUS, PATHOLOGY FEATURE	(S3h.15)
-2.77	0.0022	9	**12 o.clock superior? (. ,|and|margin of) the**		(S3h.16)
-2.37	0.0023	12	**consists of (three|a|one|two)? 3**		(S3h.17)
-1.74	0.0028	20	**in (the stroma|extent|an addendum report|black|depth|-situ|thickness|diameter|length) .**		(S3h.18)
-3.18	0.0029	6	**1 : (Right level IIb|. Right vocal cord .|Deep medial margin) .**	NODAL, SITE	(S3h.19)
-3.18	0.0029	6	**the (specimen show|carcinoma with|lesion**		(S3h.20)

Log (OR)	P	N	Pattern	Comments	Crossref.
			**appears complete and) the**		
-3.18	0.0029	6	**tissue (: No evidence of malignancy .|,) 6**		(S3h.21)
-3.18	0.0029	6	**studies :? (P16 immunohistochemistry : Negative In situ hybridisation|for high risk HPV) .**	HPV STATUS	(S3h.22)
-3.18	0.0029	6	**of (tumour|the submandibular gland|resection) are**		(S3h.23)
-3.18	0.0029	6	**of (the|resection are clear) (lesion appears complete and|right side)? of? the**		(S3h.24)
-3.18	0.0029	6	**medial tongue? . , the specimen**	SITE	(S3h.25)
-3.18	0.0029	6	**a (small|large) area**		(S3h.26)
-3.18	0.0029	6	**P16 immunohistochemistry : Negative In situ hybridisation .? (and HPV|Ventana INFORM)? ISH**	HPV STATUS	(S3h.27)
-3.18	0.0029	6	**4 (:|.) . Left**		(S3h.28)
-3.18	0.0029	6	**2A - (four possible|five) (lymph nodes|nodes) ; 2B -**		(S3h.29)
-1.92	0.003	16	**invasion is (identified|present|seen|not seen) .**		(S3h.30)
-1.92	0.003	16	**of (three|one|two)? 3 mm**		(S3h.31)
-1.49	0.0042	34	**2 mm (of extranodal spread|pale biopsy|from the deep margin|fragments|biopsies)? .**	PATHOLOGY FEATURE	(S3h.32)
-1.56	0.0044	21	**of (tumour|two lymph nodes|extranodal spread|dysplasia|soft tissue|squamous mucosa|lymphocytes|muscle|floor of mouth|thyroid|pink tissue|oral type with subepithelial stroma and skeletal muscle|malignant cells|poorly differentiated carcinoma) .**	PATHOLOGY FEATURE	(S3h.33)
-1.95	0.0044	13	**Block 6A -? one? (bisected|possible)? (two . lymph nodes|node)? (;|and) 6B-**		(S3h.34)
-1.95	0.0044	13	**HPV (studies|genotypes) :? P16 immunohistochemistry :? Negative**	HPV STATUS	(S3h.35)
-1.95	0.0044	13	**perineural space? invasion is (identified|present|seen) .**		(S3h.36)
-1.67	0.0049	17	**lateral tongue? (. , the specimen consists|aspect) of**	SITE	(S3h.37)
-1.67	0.0049	17	**mm (.|right|deep|anterior|medial|superior|thick , extending|punch biopsy) to**	TUMOUR LOCATION (Type IIIB MD)	(S3h.38)
-2.22	0.0053	11	**of skin to? a (6 x|depth of)? 3**		(S3h.39)
-2.22	0.0053	11	**There is no evidence of? (high grade dysplasia|in situ)? or? invasive**	PATHOLOGY FEATURE	(S3h.40)
-2.59	0.0058	8	**a (6 x|depth of) 3**		(S3h.41)
-2.59	0.0058	8	**sections of (the|salivary gland) 3 and 9 o.clock? (submandibular gland are unremarkable|ends)? respectively? .**		(S3h.42)
-2.59	0.0058	8	**no (invasive malignancy|dysplasia|perineural invasion|significant change) .**	PATHOLOGY FEATURE	(S3h.43)
-2.59	0.0058	8	**one (end|edge|transverse section) of the**		(S3h.44)
-2.59	0.0058	8	**of (submandibular gland|margins|resection are clear of)? the lesion? ;**		(S3h.45)
-1.60	0.006	19	**The (base|excision|central portion|focus|regions|presence|edge|remainder|outside) of**	SITE (Type IIIB MD)	(S3h.46)
-1.39	0.0071	26	**and (9 o.clock ends respectively|fatty tissue|all**	PATHOLOGY	(S3h.47)

Log (OR)	P	N	Pattern	Comments	Crossref.
			**embedded|subepithelial stroma|sinus|cartilage|lymphovascular invasion|fibrosis|plasma cells|chronic inflammation|CK5.6|hyperkeratosis|radiotherapy|mild chronic inflammation|venous invasion) .**	FEATURE	
-2.98	0.0083	5	**- (two transverse sections|longitudinal sections|shave) of**		(S3h.48)
-2.98	0.0083	5	**the closest (transverse|peripheral)? margin**		(S3h.49)
-2.98	0.0083	5	**. . (Left level 4|Larynx|Right vocal cord|Left vocal cord) .**	NODAL, SITE	(S3h.50)
-2.98	0.0083	5	**right neck dissection? :**		(S3h.51)
-2.98	0.0083	5	**of moderately differentiated , keratinising? squamous cell carcinoma**	PATHOLOGY FEATURE	(S3h.52)
-2.98	0.0083	5	**consists of (a|two pale tan biopsies ,) 4**		(S3h.53)
-2.98	0.0083	5	**are clear (of|from) the**		(S3h.54)
-2.98	0.0083	5	**the (submandibular gland|appearances) are**		(S3h.55)
-2.98	0.0083	5	**studies :? P16 immunohistochemistry : Negative**	HPV STATUS	(S3h.56)
-2.98	0.0083	5	**punch biopsy of skin? to a depth**	TUMOUR LOCATION	(S3h.57)
-2.98	0.0083	5	**both (ends|negative) .**		(S3h.58)
-2.98	0.0083	5	**and the? underlying skeletal muscle**		(S3h.59)
-2.98	0.0083	5	**3 (mm|o.clock) to**		(S3h.60)
-1.40	0.0099	22	**. (No evidence of malignancy|medial tongue|No tumour|larynx|Larynx|total laryngectomy|subtotal glossectomy|Right vocal cord|Left vocal cord) .**	SITE	(S3h.61)

Note: In Tables S3(c), (d), (g), and (h), regular expressions that group only cardinal numbers as options [e.g. *“size (15|20|30|...) mm”*] are not shown for brevity.

Table S4: Full list of features listed mined by TEPAPA (FDG-PET/CT reports)

Table S4(a): Positive binary features (n-gram) without sequence-level annotation

Log (OR)	P	N	Pattern	Comments	Crossref.
2.95	7.6×10^{-7}	34	**base**	SITE (Type IIIA MD)	(S4a.1)
4.03	1.2×10^{-6}	22	**tonsillar**	SITE	(S4a.2)
3.30	0.00049	14	**vallecula**	SITE	(S4a.3)
1.79	0.0015	30	**bowel**		(S4a.4)
2.64	0.0029	16	**tonsil**	SITE	(S4a.5)
2.54	0.0033	15	**Previous**		(S4a.6)
2.98	0.005	11	**FDG avid pulmonary nodule**	METASTATIC DISEASE (Type IIIB MD)	(S4a.7)
2.98	0.005	11	**No FDG avid pulmonary**	METASTATIC DISEASE	(S4a.8)
2.98	0.005	11	**hypermetabolism in**		(S4a.9)
2.98	0.005	11	**remaining oropharynx**		(S4a.10)
2.86	0.0051	10	**Primary site : There**		(S4a.11)
2.01	0.0054	18	**No pulmonary**		(S4a.12)
2.01	0.0054	18	**soft tissue mass**		(S4a.13)
2.43	0.0062	14	**oropharyngeal**		(S4a.14)
1.48	0.0063	46	**or pleural**		(S4a.15)
1.52	0.0068	27	**, pancreas**		(S4a.16)
1.52	0.0068	27	**lesion is detected**		(S4a.17)
1.75	0.0077	21	**pancreas ,**		(S4a.18)
1.34	0.0089	40	**tongue**	SITE	(S4a.19)
1.59	0.0092	58	**REPORT**		(S4a.20)
2.73	0.0099	9	**lesion is demonstrated**		(S4a.21)
2.73	0.0099	9	**lymphadenopathy is demonstrated**		(S4a.22)

Table S4(b): Positive binary features (n-gram) with sequence-level annotation using UMLS vocabulary

Log (OR)	P	N	Pattern	Comments	Crossref.
3.02	0.00014	20	**tonsillar**	SITE	(S4b.1)
3.30	0.00049	14	**vallecula**	SITE	(S4b.2)
2.74	0.0014	17	**base of tongue**	SITE	(S4b.3)
2.54	0.0033	15	**Previous**		(S4b.4)
1.85	0.004	22	**tongue base**	SITE	(S4b.5)
2.98	0.005	11	**loops**		(S4b.6)
2.98	0.005	11	**hypermetabolism in**		(S4b.7)
2.98	0.005	11	**FDG avid pulmonary nodule**	METASTATIC DISEASE (Type IIIB MD)	(S4b.8)
2.98	0.005	11	**remaining oropharynx**		(S4b.9)
2.86	0.0051	10	**Primary site : There**		(S4b.10)
2.01	0.0054	18	**soft tissue mass**		(S4b.11)
2.43	0.0062	14	**oropharyngeal**		(S4b.12)
1.52	0.0068	27	**, pancreas**		(S4b.13)
1.52	0.0068	27	**lesion is detected**		(S4b.14)

Table S4: Full list of features listed mined by TEPAPA (FDG-PET/CT reports) - (Cont'd)

Table S4(c): Positive binary features (regular expressions) without sequence-level annotation

<table>
<tr><th>Log (OR)</th><th>P</th><th>N</th><th>Pattern</th><th>Comments</th><th>Crossref.</th></tr>
<tr><td>3.29</td><td>$2×10^{-5}$</td><td>23</td><td>No FDG avid? pulmonary (nodules|nodule) or pleural</td><td>METASTATIC DISEASE</td><td>(S4c.1)</td></tr>
<tr><td>1.94</td><td>0.00031</td><td>35</td><td>the (body|nasopharynx|vallecula|epiglottis|retroperitoneum|skeleton|abdomen , pelvis inguinal regions|primary site|current study|oropharynx|hyoid bone|thorax|pharyngeal mucosal space|uterus|solid abdominal organs|trunk) .</td><td>SITE, TUMOUR LOCATION (Type IIIA MD)</td><td>(S4c.2)</td></tr>
<tr><td>3.20</td><td>0.001</td><td>13</td><td>avid pulmonary (nodules|nodule) or pleural</td><td>METASTATIC DISEASE (Type IIIB MD)</td><td>(S4c.3)</td></tr>
<tr><td>3.20</td><td>0.001</td><td>13</td><td>is (identified|detected) . both? on</td><td></td><td>(S4c.4)</td></tr>
<tr><td>2.29</td><td>0.0012</td><td>21</td><td>in the (right cubital fossa|body|base of tongue|skeleton|abdomen , pelvis inguinal regions|current study|thorax|trunk) .</td><td>SITE</td><td>(S4c.5)</td></tr>
<tr><td>2.74</td><td>0.0014</td><td>17</td><td>base of the? tongue</td><td>SITE</td><td>(S4c.6)</td></tr>
<tr><td>1.75</td><td>0.0016</td><td>33</td><td>pulmonary (nodules|nodule) or pleural</td><td></td><td>(S4c.7)</td></tr>
<tr><td>1.94</td><td>0.002</td><td>23</td><td>pulmonary nodule or pleural? (effusion|abnormality)? is</td><td>METASTATIC DISEASE (Type IIIB MD)</td><td>(S4c.8)</td></tr>
<tr><td>1.94</td><td>0.002</td><td>23</td><td>in the? (the|left|remaining) oropharynx</td><td></td><td>(S4c.9)</td></tr>
<tr><td>3.09</td><td>0.0023</td><td>12</td><td>lesion is (identified|detected) . both? on</td><td></td><td>(S4c.10)</td></tr>
<tr><td>3.09</td><td>0.0023</td><td>12</td><td>No FDG avid? pulmonary? (nodules|nodule)? (or pleural|new) abnormality</td><td>METASTATIC DISEASE</td><td>(S4c.11)</td></tr>
<tr><td>3.09</td><td>0.0023</td><td>12</td><td>avid (4 mm|pulmonary) nodule</td><td>METASTATIC DISEASE (Type IIIB MD)</td><td>(S4c.12)</td></tr>
<tr><td>3.09</td><td>0.0023</td><td>12</td><td>bowel (appear unremarkable|activity|loops) .</td><td></td><td>(S4c.13)</td></tr>
<tr><td>3.09</td><td>0.0023</td><td>12</td><td>right (tonsillar|tongue base|tonsil) SCC</td><td>SITE</td><td>(S4c.14)</td></tr>
<tr><td>1.79</td><td>0.0026</td><td>26</td><td>with (: >90% Probable OR Probably : approx|metastasis|altered usage|metastatic disease|central necrosis|high-grade malignancy|unknown primary) .</td><td></td><td>(S4c.15)</td></tr>
<tr><td>2.20</td><td>0.0026</td><td>20</td><td>No (FDG avid|new)? pulmonary nodule</td><td>METASTATIC DISEASE</td><td>(S4c.16)</td></tr>
<tr><td>2.54</td><td>0.0033</td><td>15</td><td>the (right|left)? base of</td><td>SITE (Type IIIA MD)</td><td>(S4c.17)</td></tr>
<tr><td>2.54</td><td>0.0033</td><td>15</td><td>No pulmonary (nodules|nodule) or</td><td></td><td>(S4c.18)</td></tr>
<tr><td>1.61</td><td>0.0037</td><td>28</td><td>the (vallecula|mediastinum|retroperitoneum|midline|mandible|abdomen|previous study|axillary|probability of that diagnosis) ,</td><td>SITE</td><td>(S4c.19)</td></tr>
<tr><td>1.61</td><td>0.0037</td><td>28</td><td>lymphadenopathy is (identified|detected|demonstrated|seen) in</td><td></td><td>(S4c.20)</td></tr>
<tr><td>1.61</td><td>0.0037</td><td>28</td><td>There is (no|an intensely|physiological|mild|marked) FDG</td><td></td><td>(S4c.21)</td></tr>
<tr><td>1.85</td><td>0.004</td><td>22</td><td>or enlarged? cervical? (lymph node|pleural) (effusion|abnormality)? is</td><td></td><td>(S4c.22)</td></tr>
<tr><td>2.98</td><td>0.005</td><td>11</td><td>the (right|left) tonsillar</td><td>SITE</td><td>(S4c.23)</td></tr>
<tr><td>2.98</td><td>0.005</td><td>11</td><td>for (staging|restaging) FDG-PET</td><td></td><td>(S4c.24)</td></tr>
</table>

<table>
<tr><th>Log (OR)</th><th>P</th><th>N</th><th>Pattern</th><th>Comments</th><th>Crossref.</th></tr>
<tr><td>2.98</td><td>0.005</td><td>11</td><td>the (neck ,|injection site)? (previous PET CT scan performed|particularly)? in</td><td></td><td>(S4c.25)</td></tr>
<tr><td>2.98</td><td>0.005</td><td>11</td><td>site (in|on) the</td><td></td><td>(S4c.26)</td></tr>
<tr><td>2.86</td><td>0.0051</td><td>10</td><td>is (identified|detected)? (. both on|complete) metabolic</td><td></td><td>(S4c.27)</td></tr>
<tr><td>2.86</td><td>0.0051</td><td>10</td><td>and (CT appearances within|in|at|inferiorly to| extends past) the</td><td></td><td>(S4c.28)</td></tr>
<tr><td>2.86</td><td>0.0051</td><td>10</td><td>spleen , (kidneys and|adrenals ,)? pancreas</td><td></td><td>(S4c.29)</td></tr>
<tr><td>2.86</td><td>0.0051</td><td>10</td><td>and bowel (appear unremarkable|loops) .</td><td></td><td>(S4c.30)</td></tr>
<tr><td>2.86</td><td>0.0051</td><td>10</td><td>No FDG avid pulmonary (nodules|nodule) or</td><td>METASTATIC DISEASE</td><td>(S4c.31)</td></tr>
<tr><td>2.01</td><td>0.0054</td><td>18</td><td>nodule or pleural (effusion|abnormality) is</td><td></td><td>(S4c.32)</td></tr>
<tr><td>2.43</td><td>0.0062</td><td>14</td><td>: No FDG avid? pulmonary</td><td>METASTATIC DISEASE</td><td>(S4c.33)</td></tr>
<tr><td>2.43</td><td>0.0062</td><td>14</td><td>in the (mediastinum|lungs , liver ,|axillae| proximal appendicular) or? (mediastinum| retroperitoneum|axial)? skeleton? or elsewhere? .</td><td></td><td>(S4c.34)</td></tr>
<tr><td>2.43</td><td>0.0062</td><td>14</td><td>for (staging|restaging)? (a progress PET CT| FDG-PET) scan</td><td></td><td>(S4c.35)</td></tr>
<tr><td>2.43</td><td>0.0062</td><td>14</td><td>in the? remaining? oropharynx , oral cavity ,? nasopharynx ,? hypopharynx</td><td></td><td>(S4c.36)</td></tr>
<tr><td>2.43</td><td>0.0062</td><td>14</td><td>the remaining? oropharynx ,</td><td></td><td>(S4c.37)</td></tr>
<tr><td>2.73</td><td>0.0099</td><td>9</td><td>: No FDG avid cervical? (lymphadenopathy| suspicious osseous lesion|new finding) is</td><td>METASTATIC DISEASE</td><td>(S4c.38)</td></tr>
<tr><td>2.73</td><td>0.0099</td><td>9</td><td>kidneys and (bowel loops|urinary bladder are of normal appearance) .</td><td></td><td>(S4c.39)</td></tr>
<tr><td>2.73</td><td>0.0099</td><td>9</td><td>No intracranial? (space|space-occupying)? (suspicious osseous|-occupying|occupying)? lesion is detected . both? on</td><td>METASTATIC DISEASE</td><td>(S4c.40)</td></tr>
<tr><td>2.73</td><td>0.0099</td><td>9</td><td>avid cervical? (lymphadenopathy|mucosal) , subcutaneous? or cutaneous lesion? is demonstrated</td><td>TUMOUR LOCATION (Type IIIA MD)</td><td>(S4c.41)</td></tr>
<tr><td>2.73</td><td>0.0099</td><td>9</td><td>avid pulmonary? (nodules|nodule|cutaneous)? or pleural? (effusion|destructive bone| subcutaneous)? lesion? is? (identified|detected| noted|effusions) .</td><td>METASTATIC DISEASE (Type IIIB MD)</td><td>(S4c.42)</td></tr>
<tr><td>2.73</td><td>0.0099</td><td>9</td><td>cutaneous or subcutaneous? lesion is (detected|demonstrated)? (identified|in the head and neck) .</td><td></td><td>(S4c.43)</td></tr>
<tr><td>2.73</td><td>0.0099</td><td>9</td><td>is (identified|detected) elsewhere? . (M0|both on metabolic and anatomic grounds) .</td><td>STAGE</td><td>(S4c.44)</td></tr>
<tr><td>2.73</td><td>0.0099</td><td>9</td><td>the (right|left|remaining) oropharynx</td><td></td><td>(S4c.45)</td></tr>
</table>

Table S4: Full list of features listed mined by TEPAPA (FDG-PET/CT reports) – (Cont'd)

Table S4(d): Positive binary features (regular expressions) with sequence-level annotation using UMLS vocabulary

Log (OR)	P	N	Pattern	Comments	Crossref.
1.94	0.00028	38	**the (body|nasopharynx|vallecula| retroperitoneum|skeleton|PET scan|abdomen , pelvis inguinal regions|right lower lobe|previous scan|current study|primary site|oropharynx|base of tongue|hyoid bone|thorax|pharyngeal mucosal space|uterus|solid abdominal organs| trunk) .**	SITE, TUMOUR LOCATION (Type IIIB MD)	(S4d.1)
2.93	0.0003	19	**avid (pulmonary nodules|lytic|pulmonary nodule| cutaneous) or**	METASTATIC DISEASE (Type IIIA MD)	(S4d.2)
3.20	0.001	13	**No FDG avid? (pulmonary nodules|pulmonary nodule) or pleural**	METASTATIC DISEASE	(S4d.3)
3.20	0.001	13	**is (identified|detected) . both? on**		(S4d.4)
2.74	0.0014	17	**, adrenal glands ,? spleen , adrenal glands ,? gallbladder ,? pancreas**		(S4d.5)
1.94	0.002	23	**in the? (the|left|remaining) oropharynx**		(S4d.6)
3.09	0.0023	12	**lesion is (identified|detected) . both? on**		(S4d.7)
3.09	0.0023	12	**No (pulmonary nodules|FDG avid)? pulmonary nodule? (or pleural|new) abnormality**	METASTATIC DISEASE	(S4d.8)
2.20	0.0026	20	**FDG avid (pulmonary nodules|lytic|pulmonary nodule|cutaneous)? or**	METASTATIC DISEASE (Type IIIA MD)	(S4d.9)
2.64	0.0029	16	**right (.|vallecula|tonsil SCC|cubital fossa) .**	SITE	(S4d.10)
1.61	0.0037	28	**lymphadenopathy is (identified|detected| demonstrated|seen) in**		(S4d.11)
1.61	0.0037	28	**There is (no|an intensely|physiological|mild| marked) FDG**		(S4d.12)
2.98	0.005	11	**No intracranial space? (suspicious osseous|- occupying|occupying) lesion is (identified| detected) . both? on**	METASTATIC DISEASE	(S4d.13)
2.98	0.005	11	**the (neck ,|injection site)? (previous PET CT scan performed|particularly)? in**		(S4d.14)
2.98	0.005	11	**for (staging|restaging) FDG-PET scan**		(S4d.15)
2.98	0.005	11	**normal (-appearing|appearing) liver**		(S4d.16)
2.86	0.0051	10	**is (identified|detected)? (. both on|complete) metabolic**		(S4d.17)
2.86	0.0051	10	**spleen , (kidneys and|adrenals ,)? pancreas**		(S4d.18)
2.86	0.0051	10	**bowel (activity|loops) .**		(S4d.19)
2.86	0.0051	10	**for (staging|restaging) FDG-PET scan .**		(S4d.20)
2.01	0.0054	18	**pulmonary nodule or (pleural effusion|pleural abnormality) is**		(S4d.21)
2.43	0.0062	14	**the remaining? oropharynx ,**		(S4d.22)
2.43	0.0062	14	**in the? remaining? oropharynx , oral cavity ,? nasopharynx ,? hypopharynx**		(S4d.23)
2.43	0.0062	14	**, in keeping with? a? (non|high) -**		(S4d.24)
2.43	0.0062	14	**No FDG avid (pulmonary nodules|pulmonary**	METASTATIC	(S4d.25)

Log (OR)	P	N	Pattern	Comments	Crossref.
			**nodule)? or**	DISEASE	
2.73	0.0099	9	**: No FDG avid cervical? (lymphadenopathy| suspicious osseous lesion|new finding) is**	METASTATIC DISEASE	(S4d.26)
2.73	0.0099	9	**FDG avid (pulmonary nodules|pulmonary nodule|cutaneous)? or (pleural effusion|pleural effusions|destructive bone lesion|subcutaneous lesion) is? (identified|detected|noted)? .**	METASTATIC DISEASE (Type IIIA MD)	(S4d.27)
2.73	0.0099	9	**FDG avid cervical? (lymphadenopathy| mucosal) , subcutaneous? or cutaneous lesion? is demonstrated**	TUMOUR LOCATION (Type IIIB MD)	(S4d.28)
2.73	0.0099	9	**kidneys and (bowel loops|urinary bladder are of normal appearance) .**		(S4d.29)
2.73	0.0099	9	**No (other|new)? pulmonary nodule or pleural? abnormality is identified**	METASTATIC DISEASE (Type IIIA MD)	(S4d.30)
2.73	0.0099	9	**is (identified|detected) elsewhere? . (M0|both on metabolic and anatomic grounds) .**	STAGE	(S4d.31)
2.73	0.0099	9	**pulmonary nodules or pleural abnormality? (or pleural effusions|are detected) .**	METASTATIC DISEASE (Type IIIA MD)	(S4d.32)
2.73	0.0099	9	**right (level IIA|retropharyngeal|tonsil SCC|tonsil| paratracheal) and**	NODAL, SITE	(S4d.33)
2.73	0.0099	9	**the (right|left|remaining) oropharynx**		(S4d.34)
2.73	0.0099	9	**the (superior|apical) segment**		(S4d.35)

Table S4(e): Negative binary features (n-gram) without sequence-level annotation

Log (OR)	P	N	Pattern	Comments	Crossref.
-3.23	0.0026	6	**on the background of**		(S4e.1)
-3.23	0.0026	6	**and anterior**		(S4e.2)
-2.64	0.0049	8	**Distant Disease :**		(S4e.3)
-3.02	0.0074	5	**which may**		(S4e.4)
-3.02	0.0074	5	**subglottic**		(S4e.5)
-1.39	0.0083	36	**intense**		(S4e.6)
-1.84	0.0088	12	**moderately**		(S4e.7)

Table S4(f): Negative binary features (n-gram) with sequence-level annotation using UMLS vocabulary

Log (OR)	P	N	Pattern	Comments	Crossref.
-3.23	0.0026	6	**on the background of**		(S4f.1)
-2.64	0.0049	8	**Distant Disease :**		(S4f.2)
-3.02	0.0074	5	**and anterior**		(S4f.3)
-3.02	0.0074	5	**infiltration**		(S4f.4)
-3.02	0.0074	5	**which may**		(S4f.5)
-3.02	0.0074	5	**subglottic**		(S4f.6)
-3.02	0.0074	5	**- No hypermetabolic/ enlarged lymph nodes are detected**	NODAL	(S4f.7)
-1.39	0.0083	36	**intense**		(S4f.8)
-1.84	0.0088	12	**moderately**		(S4f.9)

Table S4: Full list of features listed mined by TEPAPA (FDG-PET/CT reports) – (Cont'd)

Table S4(g): Negative binary features (regular expressions) without sequence-level annotation

Log (OR)	P	N	Pattern	Comments	Crossref.
-1.98	0.0014	16	**intense (hypermetabolism|increased FDG activity) .**		(S4g.1)
-2.15	0.0016	14	**is (noted without abnormal|intense) hypermetabolism**		(S4g.2)
-2.64	0.0049	8	**the (right|left|true)? vocal cord**	SITE	(S4g.3)
-2.64	0.0049	8	**hilar lymph? (nodes|node) .**		(S4g.4)
-3.02	0.0074	5	**- No (hypermetabolic.enlarged|hypermetabolic. enlarged) lymph nodes are detected**		(S4g.5)
-3.02	0.0074	5	**the (right|left|true) vocal**		(S4g.6)
-3.02	0.0074	5	**nodes (, measuring up|are considered more likely) to**		(S4g.7)
-1.84	0.0088	12	**the (anterior|lateral|inferior) aspect**	TUMOUR LOCATION (Type IIIB MD)	(S4g.8)
-1.84	0.0088	12	**anterior (to|segment|margin|aspect) of? the**	TUMOUR LOCATION (Type IIIB MD)	(S4g.9)

Table S4(h): Negative binary features (regular expressions) with sequence-level annotation using UMLS vocabulary

Log (OR)	P	N	Pattern	Comments	Crossref.
-1.98	0.0014	16	**intense (hypermetabolism|increased FDG activity) .**		(S4h.1)
-2.15	0.0016	14	**is (noted without abnormal|intense) hypermetabolism**		(S4h.2)
-2.64	0.0049	8	**anterior (to|aspect of) the**	TUMOUR LOCATION (Type IIIB MD)	(S4h.3)
-1.84	0.0088	12	**the (anterior|lateral|inferior) aspect**	TUMOUR LOCATION (Type IIIB MD)	(S4h.4)
-1.84	0.0088	12	**- (No destructive bony lesion . -|There)? (CT findings|No hypermetabolic. enlarged lymph nodes)? are**		(S4h.5)
-1.59	0.0092	16	**, (III|retroperitoneum|axillary|mesenteric|2A|oropharynx) ,**		(S4h.6)

Note: In Tables S4(c), (d), (g), and (h), regular expressions that group only cardinal numbers as options [e.g. *"size (15|20|30|...) mm"*] are not shown for brevity.

Table S5. List of features mined by TEPAPA – other variables

<table>
<tr><th>Phenotype or variable</th><th>Measure</th><th>Est.</th><th>P</th><th>N</th><th>Pattern</th><th>Crossref.</th></tr>
<tr><td>Gender: Male</td><td>LOR</td><td>6.62</td><td>1.7×10^{-13}</td><td>69</td><td>“He”</td><td>(S5.1)</td></tr>
<tr><td></td><td>LOR</td><td>4.86</td><td>8.3×10^{-9}</td><td>59</td><td>“He (presents|attends)? now? (with|is|had)? (on|will require|underwent|requires)? a”</td><td>(S5.2)</td></tr>
<tr><td>Gender: Female</td><td>LOR</td><td>8.26</td><td>1.6×10^{-15}</td><td>13</td><td>“She is”</td><td>(S5.3)</td></tr>
<tr><td></td><td>LOR</td><td>7.08</td><td>1.2×10^{-13}</td><td>12</td><td>“She is? (on|has)? (been|attends|presents)? now? with? a”</td><td>(S5.4)</td></tr>
<tr><td>Age (years)</td><td>AUC</td><td>0.84</td><td>2.7×10^{-6}</td><td>21</td><td>“at (both|the) (left|right)? lung”</td><td>(S5.5)</td></tr>
<tr><td></td><td>AUC</td><td>0.85</td><td>7.6×10^{-6}</td><td>18</td><td>“AP (view of the chest|erect|semierect)? mobile? (film|projection)? .”</td><td>(S5.6)</td></tr>
<tr><td></td><td>AUC</td><td>0.75</td><td>0.00011</td><td>44</td><td>“chronic”</td><td>(S5.7)</td></tr>
<tr><td></td><td>AUC</td><td>0.76</td><td>0.00025</td><td>22</td><td>“retired”</td><td>(S5.8)</td></tr>
<tr><td>Laterality: left</td><td>LOR</td><td>5.36</td><td>1.5×10^{-8}</td><td>16</td><td>“SCC (of|involving) the left”</td><td>(S5.9)</td></tr>
<tr><td>Laterality: right</td><td>LOR</td><td>3.96</td><td>3.3×10^{-5}</td><td>23</td><td>“SCC of the right”</td><td>(S5.10)</td></tr>
<tr><td>Node: positive</td><td>LOR</td><td>2.44</td><td>4.7×10^{-5}</td><td>41</td><td>“one of”</td><td>(S5.11)</td></tr>
<tr><td></td><td>LOR</td><td>2.05</td><td>0.00029</td><td>46</td><td>“Metastatic”</td><td>(S5.12)</td></tr>
<tr><td></td><td>LOR</td><td>2.81</td><td>0.00049</td><td>27</td><td>“extranodal”</td><td>(S5.13)</td></tr>
<tr><td></td><td>LOR</td><td>3.15</td><td>0.00094</td><td>20</td><td>“- cT2? (N1|T1|cT4)? (N2b|cT3|T3)? (N2c|cT1 N2a)? M0”</td><td>(S5.14)</td></tr>
<tr><td>Node: negative</td><td>LOR</td><td>6.36</td><td>1×10^{-15}</td><td>21</td><td>“N0 M0”</td><td>(S5.15)</td></tr>
<tr><td></td><td>LOR</td><td>2.98</td><td>7.3×10^{-5}</td><td>11</td><td>“mm thick”</td><td>(S5.16)</td></tr>
<tr><td></td><td>LOR</td><td>2.79</td><td>0.00031</td><td>10</td><td>“There is no lymphadenopathy”</td><td>(S5.17)</td></tr>
<tr><td>Recurrent disease</td><td>LOR</td><td>4.00</td><td>1.6×10^{-11}</td><td>31</td><td>“recurrent”</td><td>(S5.18)</td></tr>
<tr><td></td><td>LOR</td><td>3.33</td><td>2.7×10^{-7}</td><td>22</td><td>“is well? known to the unit”</td><td>(S5.19)</td></tr>
<tr><td></td><td>LOR</td><td>3.98</td><td>5.6×10^{-6}</td><td>13</td><td>“recurrent (SCC|squamous cell carcinoma)? (of|disease in) the”</td><td>(S5.20)</td></tr>
<tr><td></td><td>LOR</td><td>-2.13</td><td>7.7×10^{-5}</td><td>53</td><td>“M0 (,|()? (p16|P16)? positive? ()|disease)? .”</td><td>(S5.21)</td></tr>
<tr><td>Tumour site: Lip</td><td>LOR</td><td>6.96</td><td>1.3×10^{-5}</td><td>3</td><td>“SCC of the lower? lip”</td><td>(S5.22)</td></tr>
<tr><td>Tumour site: Nasal cavity</td><td>LOR</td><td>6.64</td><td>0.00033</td><td>2</td><td>“: Labelled " right inferior turbinate " , the specimen consists of”</td><td>(S5.23)</td></tr>
<tr><td>Tumour site:Oropharynx</td><td>LOR</td><td>3.45</td><td>1×10^{-8}</td><td>53</td><td>“tongue”</td><td>(S5.24)</td></tr>
<tr><td></td><td>LOR</td><td>4.16</td><td>3.3×10^{-7}</td><td>33</td><td>“tonsil”</td><td>(S5.25)</td></tr>
<tr><td></td><td>LOR</td><td>3.67</td><td>2.7×10^{-5}</td><td>26</td><td>“oropharyngeal”</td><td>(S5.26)</td></tr>
<tr><td>Tumour site: Skin</td><td>LOR</td><td>5.33</td><td>0.00013</td><td>3</td><td>“carcinoma of skin”</td><td>(S5.27)</td></tr>
<tr><td>Smoking: Current smoker</td><td>LOR</td><td>4.77</td><td>1×10^{-9}</td><td>17</td><td>“cigarettes”</td><td>(S5.28)</td></tr>
<tr><td></td><td>LOR</td><td>4.34</td><td>2.6×10^{-6}</td><td>10</td><td>“a (cigarette|heavy|current) smoker”</td><td>(S5.29)</td></tr>
<tr><td></td><td>LOR</td><td>3.60</td><td>2.3×10^{-5}</td><td>11</td><td>“Inflammatory (pathology|scarring|in nature|changes).”</td><td>(S5.30)</td></tr>
<tr><td></td><td>LOR</td><td>2.64</td><td>1.7×10^{-5}</td><td>19</td><td>“per day”</td><td>(S5.31)</td></tr>
<tr><td></td><td>LOR</td><td>-3.09</td><td>0.00011</td><td>24</td><td>“nonsmoker”</td><td>(S5.32)</td></tr>
<tr><td>Smoking: Ever smoker</td><td>LOR</td><td>3.46</td><td>6.1×10^{-6}</td><td>26</td><td>“smoker”</td><td>(S5.33)</td></tr>
<tr><td></td><td>LOR</td><td>3.18</td><td>9.8×10^{-5}</td><td>23</td><td>“cigarette”</td><td>(S5.34)</td></tr>
<tr><td></td><td>LOR</td><td>3.09</td><td>0.00011</td><td>22</td><td>“consumption”</td><td>(S5.35)</td></tr>
<tr><td></td><td>LOR</td><td>3.85</td><td>1.4×10^{-5}</td><td>21</td><td>“a reformed? (cigarette|heavy|current) smoker”</td><td>(S5.36)</td></tr>
<tr><td></td><td>LOR</td><td>-4.54</td><td>2.6e-11</td><td>24</td><td>“nonsmoker”</td><td>(S5.37)</td></tr>
<tr><td>Alcohol: Current user</td><td>LOR</td><td>3.65</td><td>6.7×10^{-5}</td><td>22</td><td>“g of alcohol? daily .”</td><td>(S5.38)</td></tr>
<tr><td>Alcohol: Never consumed</td><td>LOR</td><td>-5.36</td><td>3.5×10^{-7}</td><td>7</td><td>“is a nonsmoker and? a? nondrinker”</td><td>(S5.39)</td></tr>
</table>

The rediscovery of these concepts related to categorical clinicopathologic variables can be viewed as “positive controls”. NB: Est. Estimate; LOR: log odds ratios; AUC: area under the ROC curve.

Table S6. Numeric features mined by TEPAPA

Rediscovery of concepts related to clinicopathologic variables from the ranked patterns:

Variable	Corpus	ρ	P	N	Pattern	Crossref.
Age at diagnosis (years)	MDT	1	1.4×10^{-37}	30	**“id : <PATIENTID/> <DATE/>** *<NUMBER/>* **<DOCTOR/>”**	(S6.1)
	Path	-0.69	0.0029	16	**“depth of** *<NUMBER/>* **mm”**	(S6.2)
	PET	-0.69	0.0087	13	**“suv max** *<NUMBER/>***”**	(S6.3)
Alcohol amount (g/day)	MDT	1	$<1\times10^{-100}$	15	**“***<NUMBER/>* **g of alcohol daily”**	(S6.4)
	MDT	1	3.4×10^{-51}	7	**“consumes** *<NUMBER/>* **g of alcohol daily”**	(S6.5)
Ceased smoking (*X* years ago)	MDT	0.98	3.3×10^{-5}	8	**“***<NUMBER/>* **pack”**	(S6.6)
Last smoked (*X* years ago)	MDT	1	6.4×10^{-11}	3	**“ceasing** *<NUMBER/>***”**	(S6.7)
HPV/P16 status*	PET	1	0.02	9	**“SUV max** *<NUMBER/>* **) localised to the”**	(S6.8)
	MDT	0.81	0.0099	24	**“***<NUMBER/>* **cm”**	(S6.9)
	Path				*(None discovered)*	

* Index phenotype

Table S7. Relative computational time by pipeline variation (annotation, sequence-level sequence-level annotation, threshold selection methods).

Pipeline variations	Corpus type									
	MDT reports (N=77)		Oncology clinic letters (N=14)		Pathology reports (N=75)		FDG-PET reports (N=74)		All inclusive (N=82)	
	Est.	P	Est.	P	Est.	P	Est.	P	Est.	P
**Mean *(x reference)***	2.93		(Ref)		12.83		5.46		51.36	
**Annotation method**										
None	(Ref.)									
POSTAG	5.64	<0.001	3.04	<0.001	5.34	<0.001	2.27	<0.001	NA	
SPARSE	2.45	<0.001	1.91	<0.001	1.17	0.001	1.36	<0.001	NA	
STEM	2.14	<0.001	2.22	<0.001	2.11	<0.001	2.05	<0.001	2.18	<0.001
UMLS	0.89	0.012	1.50	<0.001	0.91	0.047	0.98	0.405	0.94	<0.001
**Post-progressing**										
None	(Ref)									
REGEXI	1.07	0.013	1.06	0.013	1.12	<0.001	1.06	<0.001	1.02	0.129
**Threshold selection**										
Dev. opt. thres.	0.99	0.259	0.99	0.004	1.03	<0.001	1.04	<0.001	0.99	0.180
Adjusted R^2	0.94		0.87		0.92		0.92		0.94	

Multiple regression analysis of the factors affecting relative computational time by pipeline variations. NB: Abbreviations: FDG-PET/CT:18F-fluorodeoxyglucose Positron Emission Tomography/ Computed Tomography; MDT: multidisciplinary team; POSTAG: Part-of-speech tagging with word lemmatization; REGEXI: regular expression induction algorithm; SPARSE: syntactic parsing; STEM: token-level annotation by word stemming using Snowball algorithm; UMLS: sequence-level annotation using Meta-thesaurus from the United Medical Language System (UMLS) version 2016 AA.

Figure S8. Average predictive performance by classifier and feature filtering threshold

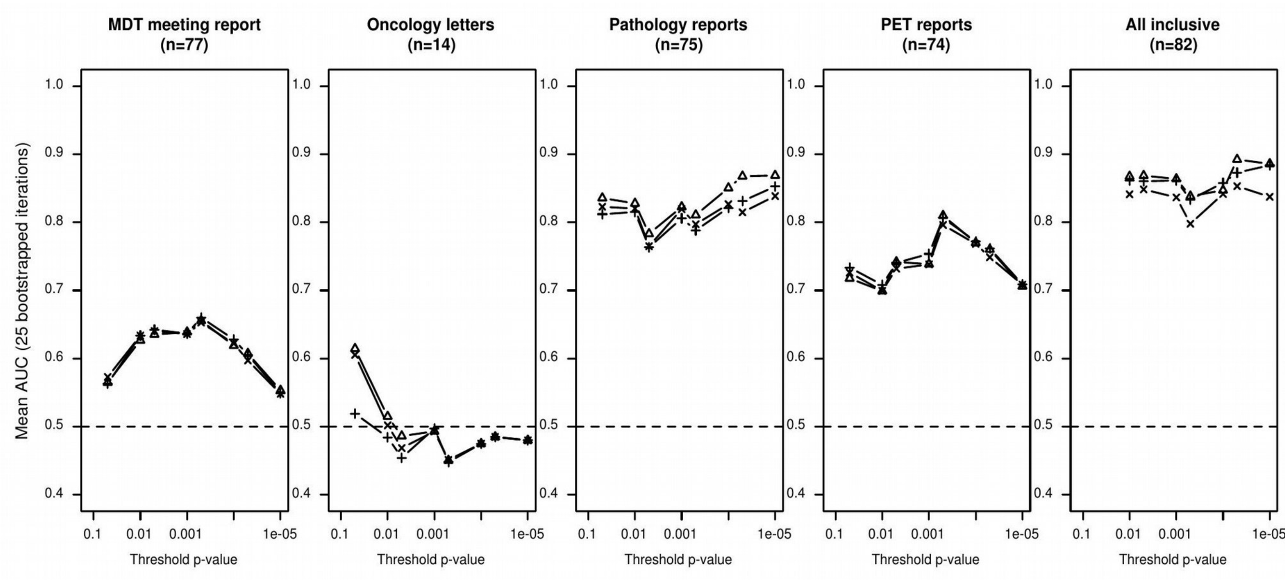

Supplement: Supplementary file 1 — Supplementary Information [file 41598_2017_7111_MOESM1_ESM.pdf]
